# A conserved loop–wedge motif moderates reaction site search and recognition by FEN1

**DOI:** 10.1093/nar/gky506

**Published:** 2018-06-07

**Authors:** Mark J Thompson, Victoria J B Gotham, Barbara Ciani, Jane A Grasby

**Affiliations:** Centre for Chemical Biology, Department of Chemistry, Krebs Institute, University of Sheffield, Brook Hill, Sheffield S3 7HF, UK

## Abstract

DNA replication and repair frequently involve intermediate two-way junction structures with overhangs, or flaps, that must be promptly removed; a task performed by the essential enzyme flap endonuclease 1 (FEN1). We demonstrate a functional relationship between two intrinsically disordered regions of the FEN1 protein, which recognize opposing sides of the junction and order in response to the requisite substrate. Our results inform a model in which short-range translocation of FEN1 on DNA facilitates search for the annealed 3′-terminus of a primer strand, which is recognized by breaking the terminal base pair to generate a substrate with a single nucleotide 3′-flap. This recognition event allosterically signals hydrolytic removal of the 5′-flap through reaction in the opposing junction duplex, by controlling access of the scissile phosphate diester to the active site. The recognition process relies on a highly-conserved ‘wedge’ residue located on a mobile loop that orders to bind the newly-unpaired base. The unanticipated ‘loop–wedge’ mechanism exerts control over substrate selection, rate of reaction and reaction site precision, and shares features with other enzymes that recognize irregular DNA structures. These new findings reveal how FEN1 precisely couples 3′-flap verification to function.

## INTRODUCTION

**Figure 1.**
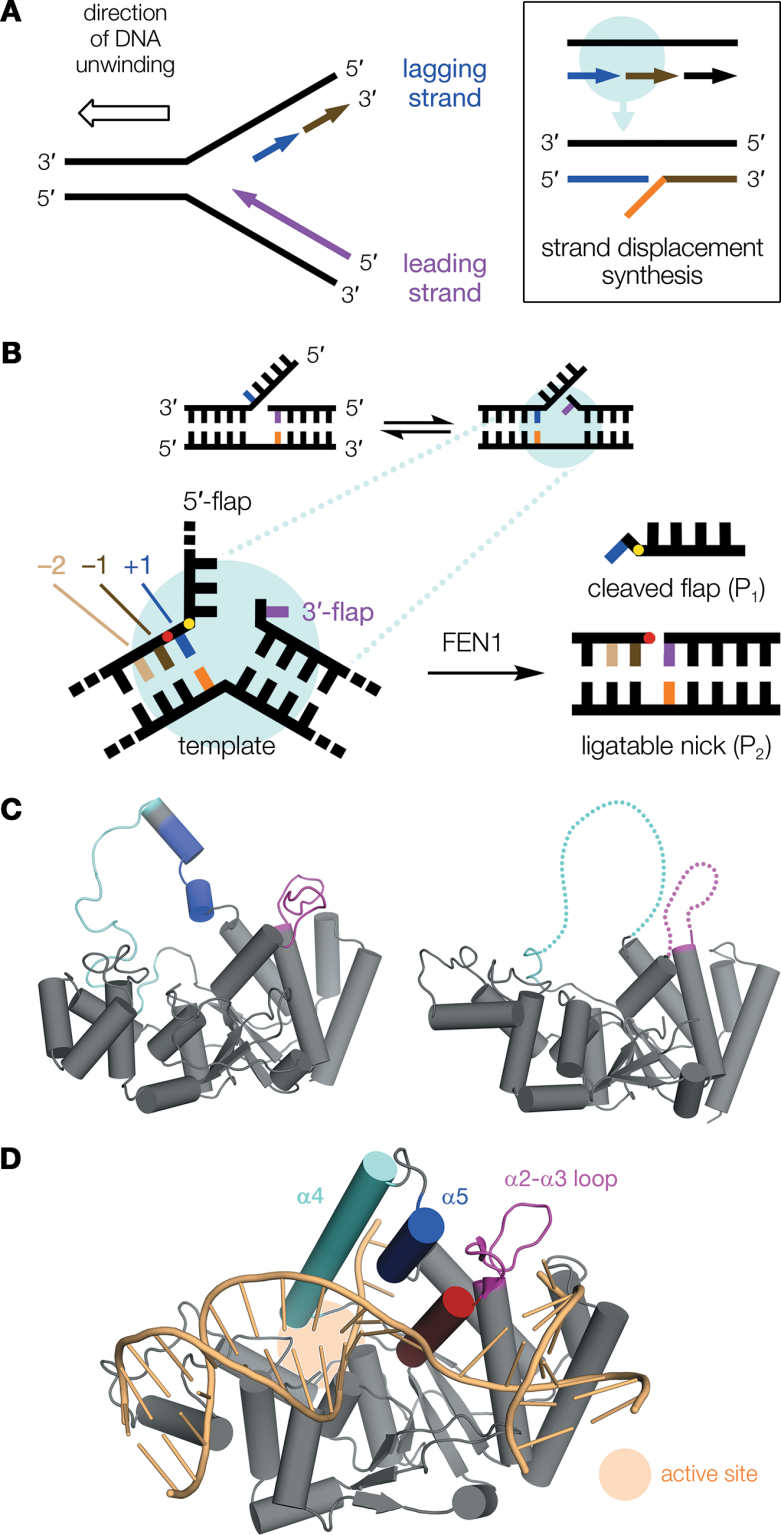
Summary of FEN1 function and structure. (**A**) During replication, the directionality of DNA polymerases (arrowheads) dictates that whereas one strand, the leading strand (purple), is synthesized continuously, the other (lagging strand) must be formed in discontinuous Okazaki fragments (blue, brown). On encountering the previous Okazaki fragment (box), synthesis continues briefly producing a displaced single-stranded DNA overhang (orange) at the junction. (**B**) These junctions theoretically exist in multiple conformers, since both stretches of newly-synthesized DNA are complementary to template. For example, the 5′-single flap conformer (left) can equilibrate to a double flap with a single nucleotide 3′-flap—the optimal FEN1 substrate (right)—because the blue and purple nucleotides are both complementary to template (orange). The detailed view shows nucleotide numbering relative to the scissile phosphate (red circle), with the +1 phosphate also indicated (yellow). (**C**) In the absence of DNA, FEN1s contain disordered regions: the α2–α3 loop (magenta) and helical arch (cyan, blue), which are mostly unstructured in *M. jannaschii* FEN1 (left; PDB code 1A76 (54)) and missing, presumed disordered (dotted lines), in hFEN1 (right; PDB code 5FV7 (55)). (**D**) hFEN1 orders upon binding substrate DNA (pale orange; PDB code 5UM9 (10)). The 5′-flap threads through the fully-formed ‘helical gateway’ (α2, red; and bottom of α4, cyan) and under the ‘helical cap’ (top of α4; and α5, blue). For clarity, this paper subsequently refers to the disordered regions only: α4–α5 as ‘arch’, and α2–α3 loop as ‘loop’.

Structure-selective nucleases (SSNs) carry out many important functions in DNA replication and repair, and their actions are essential in maintaining genome stability. For example, strand displacement synthesis during DNA replication (Figure 1A) produces intermediate structures with protruding overhangs, or flaps, which must be removed before the synthesis of new DNA can be completed by ligation. This task is carried out by flap endonuclease 1 (FEN1), a representative and well-studied SSN, which recognizes its target DNA junction structures with high selectivity (1).

Although this role in replication is the primary cellular function of FEN1, it also participates in DNA repair processes that employ strand displacement synthesis—including long-patch base excision repair (LP-BER) (2) and ribonucleotide excision repair (RER) (3)—and is rapidly recruited to sites of DNA damage in vivo (4). It is essential in mammals, and loss-of-function mutations or haploinsufficiency are both associated with elevated cancer risk (5).

The optimal substrate for FEN1 is a double-flap conformer of the overhang junction (6,7), with a single nucleotide 3′-flap (Figure 1B). The enzyme possesses a highly-conserved binding pocket for this 3′-flap, which is required for both full catalytic activity and accurate reaction site selection (8). FEN1 processing involves phosphate diester hydrolysis one nucleotide into the opposing 5′-flap duplex (red dot, Figure 1B), producing nicked DNA, itself a direct substrate for ligation to complete DNA synthesis. In the absence of a 3′-flap, FEN1 processing is slow and produces multiple products that would necessitate DNA repair prior to ligation. Thus, 3′-flap recognition is essential for the efficiency of DNA replication and repair involving FEN1.

However, it is not well understood how 3′-flap verification is coupled to function. Contacts to the 3′-flap only form a small part of the protein–DNA interface, so selection against non-3′-flap substrate conformers through differences in binding affinity alone appears unlikely, and does not explain the observed specificity. Given that the α2–α3 loop of the 3′-flap binding pocket is disordered in the absence of DNA (Figure 1C and Supplementary Figure S1; ruling out a lock-and-key type process), a mechanism wherein 3′-flap binding somehow controls interaction with another part of the substrate is more attractive.

Recognition of the opposing side of the double flap conformer—specifically the 5′-flap—is also a critical event in regulation of FEN1 activity. 5′-Flaps are threaded through a feature of the protein known as the ‘helical arch’ (α4–α5, Figure 1C and D). This serves to confine reaction to discontinuous DNA substrates and exclude continuous single-stranded DNA (ssDNA) structures, which are important intermediates in other cellular processes and must not be cut. The ‘helical arch’ region straddles the FEN1 active site and is also disordered in substrate-free protein. Based on the proximity of the α2–α3 loop and ‘helical arch’ regions of the protein (from now on referred to as ‘loop’ and ‘arch’ as defined in Figure 1D), and the observation they pack against each other when DNA is bound (Supplementary Figure S2), it is possible that these domains may be coupled (9): that 3′-flap binding induces the observed arch ordering and is therefore a pivotal event in ground-state FEN1 substrate recognition. Notably, the arch also provides a number of invariant and semi-conserved basic residues within, or close to, the FEN1 active site (10); thus, an allosteric binding model could plausibly rationalize control of reaction by 3′-flap binding distant from the active site. Consistent with this proposal, recent NMR evidence shows that changes in conformational dynamics of both the loop and arch regions occur in response to a double-flap DNA substrate (11).

Here, we present data demonstrating allosteric coupling of the FEN1 loop and arch, showing that these disordered domains are co-operatively exploited to achieve significant control over reactivity and substrate selectivity. Importantly, we identify a highly-conserved ‘wedge residue’ situated in the middle of the disordered loop, which we term a ‘loop–wedge motif’, and whose function is crucial to 3′-flap recognition in a manner reminiscent of that used by some other DNA-binding enzymes. The loop–wedge is shown to directly influence active site transfer of the target phosphodiester, over 20 Å away, remotely exerting control over reactivity and thus revealing a sophisticated—but previously unappreciated—allosteric mechanism of substrate verification by FEN1. This allosteric coupling between disordered domains represents a highly elegant regulation process and provides new understanding, at a molecular level, of how FEN1 achieves highly accurate flap processing. These new discoveries explain how the strong preference of FEN1 for double-flap structures at DNA junctions, which has long been known, relates functionally to control over reactivity; and thereby offer new insight with respect to the enzyme's mode-of-action in the cell.

## MATERIALS AND METHODS

### Plasmid constructs

Wild-type (wt) hFEN1 was expressed from the pET-28b-hFEN1-(His)_6_ vector reported previously (7). All hFEN1 mutants were generated from this construct by site-directed mutagenesis, following the protocol outlined in the QuikChange site-directed mutagenesis kit (Agilent Technologies, Inc.). Mutagenic primers (with sequences as listed in Supplementary Table S1) were purchased from Fisher Scientific, reconstituted in ultrapure water, and used as supplied. Constructs for chimeric proteins C1–C3 and AfFEN1 were generated by gene synthesis (GeneArt, Thermo Fisher Scientific) and each incorporated a C-terminal (His)_6_-tag together with 5′-NcoI and 3′-XhoI restriction sites, which were used to carry out subcloning into pET-28b. Mutants C1-L53A, C2-L54A, C3-L54A and AfFEN1-L47A were generated from the requisite template by site-directed mutagenesis, analogously to the hFEN1 mutants.

### Protein expression

All proteins were expressed in Rosetta (DE3)pLysS competent cells, grown at 37°C to an OD_600_ of 0.6–0.8 and induced with 1 mM IPTG. For hFEN1 and mutants, cultures (grown in 2 × YT media) were then incubated at 18°C for 18–24 h; or for AfFEN1 and chimeras, the cultures (grown in Super Broth) were maintained at 37°C for a further 4 h. In each case, cells were harvested by centrifugation at 6000 *g* and 4°C, washed with 1 × PBS, then resuspended in buffer IMAC-A1 (20 mM Tris pH 7.0, 1.0 M NaCl, 5 mM imidazole, 0.02% NaN_3_, 5 mM β-mercaptoethanol) supplemented with SIGMA*FAST* protease inhibitor tablets (EDTA-free, Merck) and 1 mg ml^−1^ lysozyme. Each suspension was kept on ice for 2 h then stored frozen at −20°C until further processing, as described below.

### Protein purification

All purification steps were carried out using an ÄKTA prime FPLC system at 4°C, at a flow rate of 5.0 ml min^−1^ unless stated. A detailed procedure is described below, although not all steps were used in all cases, and the exact steps used for each individual protein are specified in Supplementary Table S2. Frozen lysates were thawed on ice and homogenized by sonication. Next, 0.1 volumes of a 10% (v/v) TWEEN-20 solution, in the lysis buffer described above, were added and the mixture clarified by centrifugation at 20 000–30 000 *g* and 4°C for ≥30 min. The supernatant was loaded to a Ni-NTA column (16 × 120 mm) and washed with 5 column volumes (CV) of buffer IMAC-A1 then 5 CV of buffer IMAC-A2 (20 mM Tris pH 7.0, 0.5 M NaCl, 40 mM imidazole, 0.02% NaN_3_, 0.1% v/v TWEEN-20, 5 mM β-mercaptoethanol). Elution employed a gradient of 100% buffer IMAC-A2 to 100% buffer IMAC-B1 (250 mM imidazole pH 7.2, 0.5 M NaCl, 0.02% NaN_3_, 5 mM β-mercaptoethanol) in 1.5 CV followed by 100% IMAC-B1 for 5 CV. Pooled (His)_6_-tagged protein-containing fractions were diluted 1:1 with ultrapure water containing 20 mM β-mercaptoethanol then the solution passed through a 5 ml HiTrap Q FF column (GE Healthcare Life Sciences) to remove DNA contamination. The column was then washed with a 20 CV gradient from 0–1 M NaCl in 20 mM Tris pH 8.0, 1 mM EDTA, 0.02% NaN_3_, 20 mM β-mercaptoethanol. The flow-through, containing the protein, was diluted 1:4 with cold ultrapure water containing 20 mM β-mercaptoethanol then loaded to a HiPrep Heparin FF 16/10 column (GE Healthcare Life Sciences). The column was washed with 5 CV buffer HEP-A1 (25 mM Tris pH 7.5, 1 mM CaCl_2_, 0.02% NaN_3_, 20 mM β-mercaptoethanol) then eluted with a 20 CV gradient from 0 to 1 M NaCl in HEP-A1.

Proteins judged pure at this point by SDS-PAGE were concentrated to a volume of ≤10 ml using Vivaspin 20 centrifugal concentrator(s) (10 000 MWCO) at 3000 *g* and 4°C; exchanged into 2 × SB (100 mM HEPES pH 7.5, 200 mM KCl, 2 mM CaCl_2_, 10 mM DTT, 0.04% NaN_3_) using a HiPrep 26/10 desalting column (flow rate 10 ml min^−1^); then used to prepare final enzyme stock solutions as detailed below.

For proteins requiring further purification, fractions from the major peak were pooled and diluted by slow addition of two volumes of 3 M (NH_4_)_2_SO_4_ at 4°C. The resultant solution was loaded to a HiPrep Phenyl FF (high sub) 16/10 phenylsepharose column (GE Healthcare Life Sciences). The column was washed with 7 CV P/S-B1 (25 mM Tris pH 7.5, 2.0 M (NH_4_)_2_SO_4_, 2 mM CaCl_2_, 0.02% NaN_3_, 20 mM β-mercaptoethanol) then eluted with a 20 CV gradient from 100% P/S-B1 to 100% P/S-A1 (25 mM Tris pH 7.5, 10% v/v glycerol, 1 mM CaCl_2_, 0.02% NaN_3_, 20 mM β-mercaptoethanol). Pooled protein-containing fractions were concentrated to ≤10 ml using an Amicon stirred cell (Merck Millipore) over ice then loaded to a Sephacryl S-100 HR column (GE Healthcare Life Sciences; flow rate 0.5 ml min^−1^), equilibrated with 2 CV of 2 × SB then eluted with 4 CV of the same buffer.

Pure samples in 2 × SB were concentrated to >200 μM using a Vivaspin 20 centrifugal concentrator (10 000 MWCO) with concentration determined by *A*_280_ using the calculated ϵ_280_ for the protein. The concentration was adjusted to exactly 200 μM by addition of 2 × SB. The solution was mixed 1:1 (v/v) with cold glycerol, placed on a roller-mixer at 4°C until homogenous, then divided into 1 ml aliquots and stored as 100 μM stock at −20°C. The purity of final samples was verified by SDS-PAGE (Supplementary Figure S3).

### Oligonucleotide synthesis and DNA constructs

Oligonucleotides (Supplementary Table S3) were purchased from LGC Biosearch Technologies or Kaneka Eurogentec SA, with HPLC purification. Samples were reconstituted in ultrapure water and concentrations of stock solutions determined by UV using calculated extinction coefficients (ϵ_260_). DNA constructs (Supplementary Figure S4) were annealed in FB (50 mM HEPES pH 7.5, 100 mM KCl) by heating for 5 min at 95°C and incubating at room temperature for 30 min.

### Single-turnover rate measurements (using rapid quench-flow)

The following method was employed for measurements where ‘QF’ is indicated in Supplementary Tables S4,S5 (with the exception of trapping/blocking experiments, for which the procedure is detailed separately below). Rapid quench-flow reactions to determine maximal single-turnover rate were carried out at 37°C (or 55°C where indicated) using an RQF-63 instrument (TgK Scientific Ltd., UK), essentially as described previously (7,10,12). Working solutions of enzyme and substrate were prepared at 2 × final reaction concentration in buffer RB (50 mM HEPES pH 7.5, 100 mM KCl, 8 mM MgCl_2_, 2.5 mM DTT, 0.1 mg ml^−1^ BSA) and kept on ice until use. For each reaction, 80 μl aliquots of enzyme and substrate were mixed from separate lines and quenched after a controlled time delay of between 0.0045 and 99.041 s. The quench solution was 1.5 M NaOH containing 50 mM EDTA, except for reactions with AfFEN1, where 8 M urea containing 300 mM EDTA (pH 8.0) was used.

Reactions spanning a range of time points were conducted, then the quenched samples analysed by denaturing HPLC (dHPLC) using a WAVE system (ADS Biotec) equipped with an OligoSep Cartridge (4.6 × 50 mm), and employing fluorescence detection of the FAM label, as described previously (7). Briefly, a sample injection containing ≥300 fmol DNA was eluted with a gradient using buffers A (0.1% MeCN, 2.5 mM tetrabutylammonium bromide, 1 mM EDTA) and B (80% MeCN, 2.5 mM tetrabutylammonium bromide, 1 mM EDTA), of: 5–30% B over 1 min, 30–55% B over 4.5 min, 55–100% B over 1.6 min, 100% B for 1.4 min, 100–5% B over 0.1 min, 5% B for 2.4 min (total 11.0 min). Where multiple product peaks were observed, their combined total was considered for rate measurements.

The first-order rate constant (*k*_ST_) was derived by global fitting to the experimental data using nonlinear least squares regression in GraphPad Prism 6.05 (GraphPad Software, Inc.). One- or two-phase exponential models were considered for each dataset, with selection of the appropriate model by statistical analysis using Aikake's Information Criteria (AIC). The two-phase model was preferred in most cases (with this behaviour rationalized as explained in the main text), except for values indicated in bold in Supplementary Tables S4 and S5, where a single-phase exponential fit was used. Where single-turnover rates are graphed, error bars show standard errors from regression analysis. For all single-turnover measurements (including the trapping/blocking protocol described below), two independent experiments were carried out for each enzyme/substrate combination, as technical replicates (usually duplicates). Additional experiments were deemed necessary only when the first two runs were not in good agreement (usually judged as such if the standard error was >10% of the *k*_ST_ value).

### Single-turnover rate measurements (manual sampling)

The following method was employed for measurements where ‘BENCH’ is indicated in Supplementary Tables S4 and S5 (with the exception of trapping/blocking experiments, for which the procedure is detailed separately below). Reaction mixtures (total volume 360 μl) were prepared in 1.5 ml microcentrifuge tubes, such that all components except the enzyme were combined in 360 μl of RB with substrate at 1.11× the final required concentration. A ‘no-enzyme control’ aliquot (36 μl) was withdrawn, which was incubated, quenched and analysed in parallel. Reaction tubes were pre-incubated at 37°C (or 55°C where indicated), then reaction initiated by addition of 36 μl enzyme working stock (10 × final reaction concentration in RB, kept on ice until use). At each of an appropriate series of time points, 20 μl reaction aliquots were taken and mixed with quench solution (50 μl), which was typically 250 mM EDTA (pH 8.0), except for reactions with AfFEN1, where 8 M urea containing 80 mM EDTA (pH 8.0) was used.

Analysis of samples (dHPLC) and data fitting were carried out as described above for rapid quench-flow examples. Manual sampling experiments were usually conducted in biological duplicate (i.e. independent enzyme working stocks); and as explained above for rapid quench-flow examples, two experimental runs were deemed sufficient unless the level of agreement between them was unsatisfactory. Where data was collected using both rapid quench-flow and manual sampling techniques, at least two overlapping time points were used to verify continuity of the experimental data. For cases requiring low substrate concentrations (<1 nM, as with chimeras C2 and C3), all reaction volumes were doubled, and the 40 μl sampled aliquots quenched into 80 μl of 250 mM EDTA (pH 8.0). These modifications facilitated improved detection of peaks by dHPLC at low concentration.

### Trapping and blocking experiments (using rapid quench-flow)

Data for trapping/blocking experiments was independently collected using both rapid quench-flow and manual sampling techniques, with at least two overlapping time points chosen. For rapid quench-flow experiments, line A of the instrument was used to inject the enzyme–substrate complex solutions in buffer CaRB-SA (25 mM HEPES pH 7.5, 50 mM KCl, 2 mM CaCl_2_, 2.5 mM DTT, 0.1 mg ml^−1^ BSA), and line B was charged with MgRB-SA (25 mM HEPES pH 7.5, 50 mM KCl, 16 mM MgCl_2_, 2.5 mM DTT, 0.1 mg ml^−1^ BSA). The quench solution was 8 M urea containing 300 mM EDTA (pH 8.0).

‘Trapped’ experiments were set up as follows. In a typical example, a solution of substrate SB5,1 at 10.1 nM (2.02 × the final reaction concentration) was prepared in CaRB-SA (7.425 ml), and equilibrated at 20°C. The enzyme, hFEN1-L53D (100 μM stock, 75 μl), was added (to 2 × the final reaction concentration) and the solution mixed gently then incubated for 2 min. Next, 5 molar equivalents of streptavidin (relative to substrate) were added (2.7 μl of a 17 U/mg, 2 mg ml^−1^ stock solution) and the resulting solution incubated at 20°C for a further 5 min. A series of reactions, over a set of chosen time points and performed as technical duplicates, was then run as quickly as possible using the rapid quench-flow instrument. Before and after this sequence, a 40 μl aliquot of the ‘premix’ solution was taken and mixed into 100 μl of quench, as ‘start’ and ‘end’ controls.

Analysis of all samples was then carried out by dHPLC, using a modified gradient: 5–30% B over 1 min, 30–55% B over 4.5 min, 55–100% B over 1.6 min, 100% B for 2.9 min, 100–5% B over 0.1 min, 5% B for 2.4 min (total 12.5 min). Data for the ‘control’ rate (no streptavidin) was obtained by repeating the procedure but adding an equivalent volume of CaRB-SA instead of the streptavidin stock. Where ‘blocked’ time points were required by the rapid quench-flow method (wt hFEN1 only), this was achieved by repeating the protocol but switching the order of addition/incubation steps of enzyme and streptavidin. Usually, the ‘start’ and ‘end’ controls indicated no, or negligible, reaction of the substrate in the Ca^2+^ buffer over the time taken to run the rapid quench-flow reactions.

Where significant cleavage was seen, in the case of wt hFEN1 and chimera C3 (∼10–20% reaction in trapped and control samples), the experiment was repeated but storing the premix solution on ice after taking the ‘start’ control sample. This reduced unwanted reaction in the premix significantly, to an acceptable level, without affecting the results. After dHPLC analysis, data was processed as described in the following section. Where differing enzyme and/or substrate concentrations were used (Supplementary Table S5), quantities/volumes in preparing the premix solutions were adjusted accordingly. With chimeras C2 and C3, all volumes were increased, as above, due to the low substrate concentrations required.

### Trapping and blocking experiments (manual sampling)

Working stocks of enzyme and streptavidin (5 molar equivalents relative to substrate) were prepared at 10 × final reaction concentration in CaRB-SA, and kept on ice until use. Six solutions of substrate at 1.25 × the final reaction concentration in 1.25 × CaRB-SA (total volume 144 μl) were prepared at 20°C; then blocked, trapped and control reactions each run in duplicate.

For blocked reactions, streptavidin working stock (18 μl) was added to the tube followed by incubation for 5 min; enzyme working stock (18 μl) was then added, and incubation continued for 2 min. After equilibration for ≥1 min at 37°C, a 20 μl control aliquot was withdrawn and mixed with 50 μl quench (8 M urea, 80 mM EDTA pH 8.0). Reaction was initiated by addition of 160 μl MgRB-SA, pre-warmed to 37°C. At a series of appropriate time points, 20 μl aliquots were taken and quenched as for the control aliquot.

For trapped reactions, the procedure was repeated but the order of addition/incubation of enzyme and streptavidin was switched. Control reactions were performed as for the trapped case, except that CaRB-SA was added instead of streptavidin stock. Analysis by dHPLC was carried out as for the rapid quench-flow samples.

**Figure 2.**
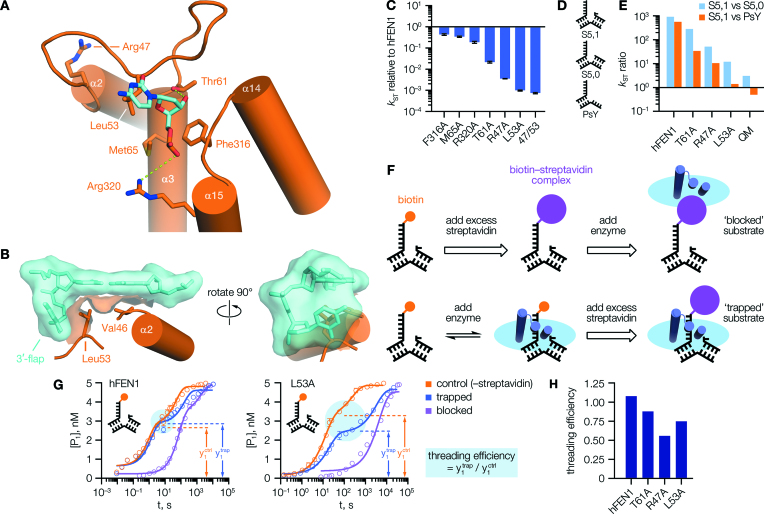
3′-Flap binding mutations impaired FEN1 activity and threading stability. (**A**) Interactions made with the 3′-flap in an hFEN1–DNA complex (PDB code 5UM9 (10)). (**B**) Partial surface render of the same structure, showing the sidechain of Leu53 positioned as a ‘wedge’ contacting both the 3′-flap base, and the terminal base pair of the ‘upstream’ duplex. (**C**) Mutation of residues shown in (A) affected FEN1 single-turnover activity to varying degrees. (**D**) Structures of DNA constructs. (**E**) Mutations affecting 3′-flap binding impaired the enzyme's selectivity. (**F**) Schematic of threading assay, illustrating how formation of a streptavidin–biotin complex at the 5′-flap terminus was used to ‘lock’ DNA substrate in a threaded or unthreaded state. To prevent reaction, complexes were formed in Ca^2+^ buffer instead of Mg^2+^. (**G**) With hFEN1 (left), the preformed ‘trapped’ complex decayed to the same initial endpoint (cyan circle) as free substrate on addition of Mg^2+^, implying the labelled substrate was fully threaded at equilibrium. With hFEN1-L53A (right), these reactions reached different endpoints indicating some substrate was unbound/unthreaded at equilibrium. The apparent hFEN1 ‘control’ rate was slowed by the requirement to displace Ca^2+^ by Mg^2+^ here; see Supplementary Figure S7C and D for details. (**H**) Threading efficiency derived for various proteins. In (C), *N* = 2, *n* = 4 (F316A, M65A, R320A, L53A quench-flow phase); *N* = 2, *n* = 6 (T61A, R47A); *N* = *n* = 4 (L53A manual sampling phase). In (**G**), *N* = 2, *n* = 4 (quench-flow phase); for manual sampling phase, *N* = *n* = 4 (L53A) or 6 (hFEN1). Ratios in (E, H) are derived from data detailed fully in Supplementary Tables S4 and S5. Error bars show SEM.

Combined data from both techniques was then fitted globally, for each enzyme/substrate combination. This followed the procedure described above for single-turnover rate determination, except that a two-phase model was always applied to the trapped dataset, and a one-phase model to the blocked. The calculated ‘threading efficiency’ was then derived, as the ratio of %_fast_ values obtained from the two-phase fit of the trapped and control reactions (equivalent to the ratio of y_1_ values, as marked on Figure 2G). Where kinetic data is graphed, error bars show SEM. Where rate values are plotted, error bars report standard errors from regression analysis.

### Multiple turnover reactions

These experiments were carried out by manual sampling. Reaction mixtures were set up and initiated as described for manual single-turnover reactions above, except with total volume scaled down to 180 μl ([S] <100 nM) or 45 μl ([S] ≥100 nM). Reactions were sampled at seven time points (2, 4, 6, 8, 10, 12 and 20 min), and 20 μl aliquots quenched into 50–200 μl of 250 mM EDTA (pH 8.0). At least 12 substrate concentrations were used (between 1 and 10 000 nM), and in each case, enzyme concentrations were chosen to give approximately 15% cleavage after 20 min; any data points showing greater conversion were discarded due to effects of substrate depletion. After dHPLC analysis as above, initial rates (*v*_0_, min^−1^) were determined by linear regression and converted to normalized rates, *v*_0_/[*E*]_0_, to adjust for enzyme concentration. Replicate datasets were fit globally—in GraphPad Prism 6.05—to the Michaelis–Menten model, with automatic outlier elimination and weighting (1/*Y*^2^) applied.

### Protein CD spectra

Aliquots of protein preparations (100 μM glycerol stocks) were diluted 1:1 with ultrapure water, on ice, then exchanged into CD buffer (1.0 mM potassium phosphate pH 7.4, 8 mM MgSO_4_, 0.1 mM EDTA, 30.7 mM (NH_4_)_2_SO_4_, 0.25 mM tris(hydroxypropyl)phosphine) using Micro Bio-Spin 6 columns (Bio-Rad) at 4°C, according to the manufacturer's protocol. Protein concentrations were quantified by UV, using calculated extinction coefficients (ϵ_280_), and the solutions adjusted to 3 μM final concentration with the same buffer. Spectra were recorded in a 1 mm path length cell between 260 and 185 nm (step 0.5 nm, time-per-point 0.5 s, bandwidth 2.0 nm, *n* = 3) using a Chirascan Plus instrument (Applied Photophysics Ltd., UK) at 20°C. For AfFEN1, spectra were also recorded at 55°C. For each protein/temperature combination, aggregated data from three independent experiments (i.e. *N* = 3, *n* = 9) was used for analysis in Pro-Data Viewer 4.4.2.0 (Applied Photophysics Ltd., UK).

Spectra were baseline-corrected, averaged, smoothed (window size = 5), and converted to units of mean residue ellipticity (MRE) to give the plots shown (Supplementary Figure S5). The spectra were analysed to extract α-helix content for each protein, using the DichroWeb (13,14) server and employing the CDSSTR (15–17) algorithm. Analysis was performed independently with each applicable reference set (sets 3, 4, 6, 7 (17,18); SP175 (19) and SMP180 (20)), and the percentage α-helix values annotated in Supplementary Figure S5 report ensemble mean values, with SD, arising from this analysis.

### Exciton-coupled CD (ECCD) assay

Samples contained 10 μM DNA substrate and 12.5 μM protein (or ‘blank’ enzyme storage buffer, i.e. 2 × SB mixed 1:1 v/v with glycerol) in buffer CaRB (50 mM HEPES pH 7.5, 100 mM KCl, 10 mM CaCl_2_), at a final glycerol concentration of 6.25% (from protein storage buffer) and a total volume of 500 μl. Then, ‘Ca^2+^’ spectra were recorded at 20°C (unless indicated otherwise), in a 5 mm path length cell, on the Chirascan Plus instrument, between 480 and 300 nm using the following settings: step 0.75 nm (480–380 nm), 0.5 nm (380–300 nm); bandwidth 2.0 nm; time-per-point 0.5 s; *n* = 2. The quality of the baseline was sensitive to inadequate sample equilibration, such that any scans with aberrant baselines were rejected. Samples were re-scanned if necessary. A 25 μl aliquot of 500 mM EDTA (pH 8.0) was then added (final EDTA concentration 23.8 mM), the sample mixed carefully, then re-scanned as above to provide ‘EDTA’ spectra. Processing was carried out using Pro-Data Viewer 4.4.2.0. All spectra were baseline-corrected by subtracting a ‘blank’ recorded with CaRB containing FB (in place of DNA substrate) and ‘blank’ enzyme storage buffer (as above, in place of enzyme), with or without EDTA, as appropriate. Corrected scans were averaged and smoothed (window size = 10), then the data plotted as Δϵ per mol 2AP residue (21). Where graphed, plots were prepared in GraphPad Prism 7.03 and are representative of the number of independent experiments specified in each case. Peak heights at 326 nm (downstream substrates) or 330 nm (upstream substrates) were used to quantify the extent to which different proteins are able to induce DNA conformational change. Where reported, these are expressed as percentages (with SEM). The value for each replicate was calculated relative to a substrate sample recorded on the same experimental run. This approach was adopted to control for variations in peak height seen between different batches of substrate/oligo which could confound analyses based on absolute Δϵ values.

### Sequence analysis

All FEN1 sequences annotated as reviewed were retrieved from the UniProt database (22), then separated by taxonomy (archaea, *n* = 51 and eukaryotes, *n* = 128; date accessed 5 June 2015). Similarly, reviewed bacterial DNA polymerase I (*polA*) sequences were retrieved (*n* = 33; date accessed 16 September 2017) and truncated to the 5′-3′ exonuclease domain (FEN1 equivalent peptide) only. Multiple sequence alignment was carried out for each set in ClustalX 2.1 (23), then the region corresponding to hFEN1 residues 34–61 was extracted and realigned, with manual adjustments made where necessary. The output from this analysis was used to generate sequence logos (24) with WebLogo 3 (25).

### Statistical analysis

Correlation analysis was carried out in GraphPad Prism 7.03, assuming Gaussian distributions and calculating Pearson correlation coefficients. Other statistical comparisons used t-tests performed in Microsoft Excel, assuming two-tailed distributions and unequal variance. In each case, detailed results are included in the relevant Figure legends. Where comparisons are made, the level of statistical significance is denoted in the appropriate Figure panels by asterisk(s) according to the following limits: *P* < 0.05 (*), <0.01 (**), <0.001 (***), <0.0001 (****).

## RESULTS

### Efficient FEN1 reaction requires loop residue Leu53

We employed site-directed mutagenesis of hFEN1 to gain insights into the functional significance of 3′-flap binding. When bound to the enzyme (Figure 2A) (10,26), the 3′-flap nucleotide is contacted by four residues: Leu53, Thr61, Phe316 and Met65. The first three of these, alongside Arg320 (which contacts the first phosphodiester of the 3′-flap strand), were previously identified as conserved and important for activity (8) but studied only as part of combination mutants such that their individual contributions, and mechanistic roles, were not known. We also included loop residue Arg47 in our analysis. It does not contact the 3′-flap directly, but is part of the arch–loop interface (Supplementary Figure S6), and the detrimental impact of R47A upon FEN1 reaction rate provided earlier circumstantial evidence suggestive of inter-domain co-operation (26,27). All these residues were mutated to alanine, either individually or in combination. Notably, during analysis of available X-ray structures we had discerned a potentially important role for Leu53, whose sidechain is consistently positioned as a ‘wedge’ guiding the 3′-flap nucleotide into its binding pocket (Figure 2B), suggestive of a key role in recognition.

Mutant enzymes were assessed using single-turnover kinetics experiments (enzyme in excess), as described previously (7,10), with an idealised double-flap FEN1 substrate that exists in a single conformer of the DNA junction (as depicted in Figure 1B, where purple and orange bases are non-complementary). This substrate is denoted S5,1 in accordance with its structure (S = ‘static’, with 5 nucleotide 5′-flap and 1 nucleotide 3′-flap), and carried a 6-carboxyfluorescein (FAM) label at the 5′-flap terminus, enabling analysis of quenched reaction aliquots by HPLC (full structural/sequence information for all substrates is given in Supplementary Figure S4 and Supplementary Table S3). Single turnover data was fit to a two-phase exponential model, and unless stated, only the initial (‘fast’) phase (usually >60%) considered in rate measurements (*k*_ST_). This is because single-turnover reactions with FEN1 proteins often display two-phase behaviour, as has been noted before (7), with the slow phase assumed to result from unproductive binding events where dissociation and rebinding are necessary for reaction (for more details, see the Methods section).

The individual mutations affected reactivity to different degrees (Figure 2C), with M65A and F316A showing little impact upon rate (*k*_ST_) compared to hFEN1, and only a small compromise for R320A. A more marked effect was seen for T61A, which removes a hydrogen bond to the 3′-OH group of the nucleotide resulting in a 50-fold reduction in activity. A 300-fold rate decrease for R47A confirmed the importance of Arg47; but significantly, L53A was the most damaging single mutation of all (1000-fold lower activity), offering apparent confirmation of the analysis above and suggesting Leu53 makes the most important contact to the 3′-flap. Indeed, combining L53A with additional mutation(s) produced little further defect in reactivity (Supplementary Figure S7A and B); and intriguingly, the R47A/L53A double mutant was no worse than L53A (Figure 2C), suggesting that if Arg47 does act as an allosteric mediator, it is transmitting information sensed first by Leu53 (noting their relative positioning; Figure 2A and Supplementary Figure S6A,B).

As noted previously (7,28), substrates lacking the 3′-flap—such as the 5′-single-flap (S5,0), or pseudo-Y (PsY) constructs (Figure 2D)—are processed hundreds of times slower compared to the double flap substrate (S5,1; Supplementary Table S5). These results underscore the importance of the 3′-flap for rapid decay of a productive enzyme–substrate complex. We rationalise the slow reaction of these substrates by understanding the enzyme–DNA complex as a dynamic conformational ensemble, and presuming it must transiently sample a catalytically-competent state with a low, but finite, probability even in the absence of key recognition contacts (i.e. 3′-flap interactions) that would normally promote rapid ‘switching’ to the reactive state. In fact, mutation of residues important for 3′-flap recognition significantly compromised the high selectivity of hFEN1 for double-flap over 5′-single-flap (S5,0) or pseudo-Y (PsY) substrates (Figure 2E and Supplementary Table S5). In the acute case of the quadruple mutant (L53A/T61A/M65A/F316A), all three substrates were processed at very similar rates, although mutation of Leu53 to alanine was alone sufficient to curtail most of the wild-type specificity, revealing the central role of the contacts made by this ‘wedge residue’ in controlling hFEN1 reactivity.

### Loop mutations affect 5′-flap threading efficiency

It is known that significant conformational rearrangements of both enzyme and substrate are required for normal FEN1 function (29–31), so we moved on to investigate the role of 3′-flap binding in these processes. As explained by an earlier disorder–thread–order model (31), threading of the 5′-flap of substrate through the arch is crucial to FEN1 activity, but only feasible through the wider aperture of a disordered arch. After threading, ordering occurs—a step proposed (9,27) to be regulated by 3′-flap binding—thus stabilizing the complex, and permitting access to the catalytically-competent state. Importantly, a properly-ordered arch is known to be required for efficient access to this catalytic state (10,12). To systematically evaluate these ideas, we adapted an existing threading assay (31) (Figure 2F) that uses a static double-flap substrate labelled with biotin at the 5′-flap terminus (denoted SB5,1). Since a streptavidin–biotin complex is much too large to fit through the FEN1 arch, threading can either be prevented (‘blocked’ case) or rendered irreversible (‘trapped’ case), depending on the order of addition of reagents.

For hFEN1, the profile of the trapped reaction matched that of the free substrate (control reaction with streptavidin omitted) in the initial phase, indicating the substrate had been fully bound and threaded at equilibrium (Figure 2G, left). The blocked reaction was considerably slower, as expected. In contrast to hFEN1, mutant L53A showed a difference in initial phase endpoints (y_1_ values; Figure 2G, right) with less product formation in the trapped case compared to control, signifying the substrate was not all bound in a threaded state at equilibrium.

By comparison of the y_1_ values for the control and trapped reactions (Figure 2G) in analogous experiments, an estimate of ‘threading efficiency’ (between 0, unthreaded; and 1, fully threaded) was made for a wider set of enzymes (Figure 2H and Supplementary Figure S7) using regression parameters obtained from the two-phase exponential fits. The 3′-flap binding mutants T61A, R47A and L53A all exhibited threading defects suggesting these residues must somehow stabilise the threaded state even though their location means they cannot be directly involved in 5′-flap threading. Recalling the disorder–thread–order model, if initial encounter leads to a complex where the 5′-flap of the substrate passes through the disordered arch, a subsequent ordering step resulting in additional arch–DNA contacts would prevent unthreading, producing a longer-lived complex (able to proceed to the reactive state). If this step is impeded, the complex is not as stable and more liable to dissociate. Thus, our results with the mutants are consistent with allosteric ‘triggering’ of arch ordering by 3′-flap recognition, provided it is assumed that the mutations do not substantially destabilise initial encounter complex formation (*k*_on_), and equilibrium threading defects are primarily attributable to raised dissociation rates (*k*_off_).

Our threading results (Supplementary Figure S7E and F) supported this assumption, because a consistent trend was apparent in the trapped case. When fitted to a two phase association model, the ‘fast’ phase (corresponding to reaction of bound-and-threaded substrate) always matched the rate of the ‘control’ reaction (free substrate). Since the reactivity of mutants—such as R47A or L53A—towards an ideal FEN1 substrate was not improved by tethering the substrate on the enzyme, we concluded that their low reactivity (as measured by *k*_ST_) is not attributable to changes in association rate (*k*_on_); and, moreover, cannot be explained by a failure to achieve a threaded state *per se*. The corresponding ‘slow’ phase of each fit matched the blocked rate, showing as expected that any substrate not bound and threaded at equilibrium became blocked upon addition of streptavidin. It should be noted that similar results were obtained with the 5′-single-flap (SB5,0) and pseudo-Y (PsY-B) substrates that lack a 3′-flap (Supplementary Figure S7G and H), which both demonstrated threading defects (indicating that a threaded, ordered complex is not so readily formed without a 3′-flap), and also gave close agreement between ‘fast’ and ‘control’ rates.

### 3′-Flap binding influences active site transfer and thereby controls reactivity

**Figure 3.**
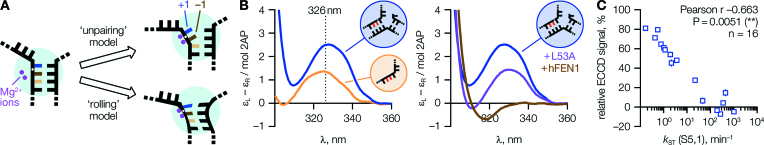
Proper positioning of the substrate for reaction relies upon 3′-flap detection. (**A**) Models for active site transfer of the scissile phosphodiester (white dot). (**B**) Left, exciton-coupled CD (ECCD) spectra of single- or double-stranded DNA containing tandem 2-aminopurine bases (2AP, red) show a maximum at around 326 nm due to exciton coupling between stacked 2APs; right, binding of a labelled substrate (DOWN-S-DF1) to hFEN1 reduces this signal to near zero, but the mutant L53A cannot induce the same extent of DNA conformational change implying that 3′-flap binding affects active site rearrangements important for catalysis. (**C**) The ability of hFEN1 3′-flap binding variants to induce this change directly correlates with catalytic activity: Pearson *r* = –0.663 (95% CI = –0.872 to –0.249, *R*^2^ = 0.440, *P* = 0.0051). ECCD spectra are representative of *N* = 37 (ssDNA), 42 (DOWN-S-DF1), 4 (+L53A) or 13 (+hFEN1) independent experiments. The correlation graph in (C) summarizes data detailed fully in Supplementary Tables S5 and S6. Error bars show SEM.

Although threading of the 5′-flap is a prerequisite for FEN1 reaction, further conformational changes of the bound DNA are still required to bring the scissile phosphodiester into contact with the catalytic metal ions—a process we term ‘active site transfer’ (29) (Figure 3A). Supported by crystallographic evidence, an unpairing model was originally proposed for this (26,30,32), wherein the two terminal base pairs of the reacting duplex are broken, separating the target strand into the active site. More recent structural results (10), with an hFEN1–DNA complex organised appropriately for reaction, are instead suggestive of ‘twisting’ or ‘rolling’ of the substrate to attain the reactive state. This state is stabilised by a number of basic residues from the arch, and therefore while its formation may require a degree of conformational flexibility, final positioning for reaction requires a structured arch (10) as noted above. Whatever the precise nature of active site transfer, significant distortion of the DNA duplex at the reaction site is clearly necessary, and this may be monitored spectroscopically using an exciton-coupled circular dichroism (ECCD) assay we have reported previously (12,29,32).

Incorporation of neighbouring 2-aminopurine (2AP) bases into DNA acts as an informative probe for local DNA conformational changes (33), since the intensity of the ECCD signal arising from this exciton pair is highly sensitive to the relative orientation of the two bases. When 2AP is placed at the −1 and −2 positions of the reacting (‘downstream’) duplex of a static, double-flap FEN1 substrate (as in DOWN-S-DF1), the expected ECCD peak is observed but reduced to near-zero on addition of hFEN1 (29,32) (in the presence of Ca^2+^ rather than Mg^2+^ to prevent reaction; Figure 3B and Supplementary Figure S8A and B), indicative of substantial DNA conformational change induced by the enzyme. The 3′-flap binding mutant L53A was unable to produce the same change (Figure 3B and Supplementary Figure S9B), showing only a partial reduction in signal. The preceding results suggest this compromised active site transfer arises from inefficient formation of the threaded and correctly ordered state; or put differently, that this state must be present with a lower population and/or lifetime in the case of the mutant.

If the average ECCD peak height (at 326 nm)—relative to substrate—was plotted against rate for the full set of 3′-flap binding mutants, a statistically significant correlation was seen (Figure 3C) which was not reflected in parallel results for a set of active site mutants (Supplementary Figure S8C). A second dataset obtained with a related substrate, incorporating 2AP at the +1 and −1 positions (DOWN-S-DF2), showed exactly the same trends (Supplementary Figure S8D–I). Thus, our ECCD results support the concept of a ‘flow’ of important functional information from the 3′-flap binding site to the active site; or in other words, the observed trends (Figure 3C) reflect a progressively lower population/lifetime of the threaded-and-ordered state as contacts with the 3′-flap are worsened, consistent with a functional allosteric relationship.

### FEN1 induces the substrate 3′-flap conformer through the Leu53 wedge

Although our preceding experiments used static substrates for clarity, flap structures formed *in vivo* as a consequence of strand displacement synthesis will potentially exhibit conformational heterogeneity, as both flaps are complementary to the template DNA strand (as shown in Figure 1B). Substrates that possesses this conformational heterogeneity are processed by FEN1 to yield the same nicked DNA product as idealized static variants. We therefore carried out ECCD measurements with 2AP probes placed in the ‘upstream’ (3′-flap) arm of such substrates, in particular looking to explore whether binding to hFEN1 generates a single nucleotide 3′-flap conformer of its substrate, as has been suggested (27,34).

**Figure 4.**
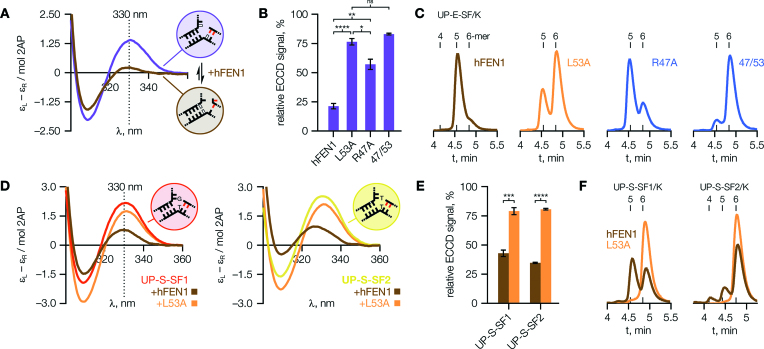
FEN1 can induce a 3′-flap. (**A**) ECCD spectra of ‘equilibrating’ substrate UP-E-SF (2APs red; white bases complementary) show a shift in the major conformational state, from single flap to double flap, upon addition of hFEN1. (**B**) The mutants shown cannot efficiently induce/stabilise this conformational change. *P* values are: 8 × 10^–8^ (hFEN1 versus L53A), 0.0044 (hFEN1 versus R47A), 0.024 (L53A vesus R47A). (**C**) HPLC analysis of reactions with UP-E-SF/K (5′-FAM labelled UP-E-SF) supports the assignment of ECCD spectra assumed above, and confirms the predominant role of wedge residue Leu53 in promoting conformational change. Expected products from UP-E-SF/K are 6-mer from the single-flap conformer and 5-mer from the double-flap conformer. Retention times of 5′-FAM labelled 4-, 5- and 6-mer standards are indicated. (**D**, **E**) With ‘static’ 5′-single-flap ECCD substrates UP-S-SF1 (left) and UP-S-SF2 (right), hFEN1 binds some in the double flap form despite a forced mismatch at the +1 position. However, mutant L53A cannot properly reconfigure either substrate. *P* values are: 0.00012 (UP-S-SF1), 2.2 × 10^–6^ (UP-S-SF2). (**F**) HPLC analysis of UP-S-SF1/K and UP-S-SF2/K reactions supports the foregoing analysis of ECCD data. ECCD spectra shown are typical of *N* = 5 (A) or 4 (D) independent experiments. HPLC chromatograms are representative of *N* = 2 (C) or 4 (F) replicates, and the reaction conditions under which they were obtained are detailed in Supplementary Table S7. In (B), *N* = 5 (hFEN1), 6 (L53A) or 3 (R47A, 47/53); and in (E), *N* = 4. Error bars show SEM.

Substrate UP-E-SF (Figure 4A and Supplementary Figure S4N) can exist in two distinct conformers, as shown, but its ECCD spectrum suggested the majority was present in the single-flap form in solution (Supplementary Figure S9A). When presented to hFEN1 its ECCD signal was sharply reduced, indicating a shift in relative orientation of the 2AP bases consistent with rearrangement to the double-flap form. This conformational rearrangement was not promoted so efficiently by mutants R47A or, especially, L53A (Figure 4B) implying that the terminal base pair of the upstream duplex is broken on binding to hFEN1, producing the stably-bound conformer through a mechanism requiring the Leu53 wedge.

### The loop–wedge 3′-flap interaction controls reaction site selection in hFEN1

HPLC analysis of reactions carried out with a 5′-FAM labelled construct (UP-E-SF/K, Supplementary Figure S4R) showed hFEN1 produced the 5-mer product expected from action on the double-flap conformer (Figure 4C). In contrast, L53A produced mostly 6-mer product, characteristic of reaction of the 5′-single-flap conformer (Figure 4A, purple circle) and consistent with the ECCD data above. Since R47A was less affected, still giving predominantly 5-mer product, these results support the suggestion above that although Arg47 is an important residue in allosteric signalling, its role is to relay structural information detected first by Leu53.

When presented with a related ‘static’ (non-equilibrating) 5′-single-flap substrate (no 3′-flap), hFEN1 also induced a change in the ECCD spectrum (Figure 4D) revealing that, surprisingly, a significant proportion of the substrate was bound in the double-flap form despite the necessity of a forced mismatch at the +1 position (as in Figure 4A, but white bases non-complementary). This observation was true of either a T–G (UP-S-SF1) or T–T (UP-S-SF2) mismatch; and as before, the ability to form the double-flap conformer required Leu53 (Figure 4E and F). These results offered additional evidence that hFEN1 generates the preferred reactive conformer of its substrate, in a manner which depends upon wedge residue Leu53.

### Domain swaps provide functional evidence for inter-domain allostery

**Figure 5.**
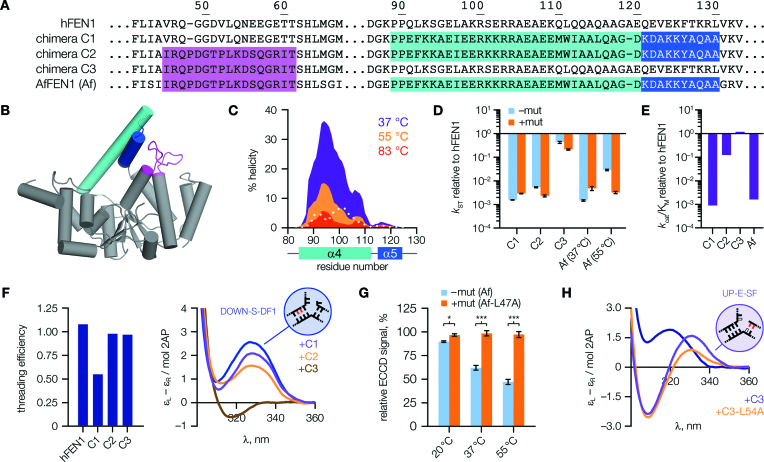
Chimeric FEN1 proteins display altered properties. (**A**) Sequence alignment of hFEN1, *Archaeoglobus fulgidus* FEN1 (AfFEN1), and human–archaeal chimeras (C1–C3). Archaeal domain(s) swapped into the human protein are highlighted in colours matching the corresponding residues in the AfFEN1 structure (**B**, made using PDB code 1RXW (9)). (**C**) AGADIR output of predicted α-helicity of the AfFEN1 arch region. Values for equivalent hFEN1 residues at 37°C are overlaid (dotted white line). (**D**) Equivalent mutations to hFEN1-L53A in the chimeras and AfFEN1 generally showed little effect. Rates are plotted relative to hFEN1, without (–mut) or with (+mut) the mutation. (**E**) Second-order rate constants, *k*_cat_/K_M_, plotted relative to hFEN1. (**F**) Left, threading efficiencies of chimeras; right, only chimera C3 can efficiently position substrate DOWN-S-DF1, even though both C2 and C3 can thread the 5′-flap fully. (**G**) With AfFEN1, the ECCD signal decreases at elevated temperatures, but this change is dependent upon contacts with the leucine ‘wedge’ (AfFEN1-Leu47 aligns with hFEN1-Leu53). *P* values are: 0.0139 (20°C), 0.00065 (37°C), 0.00030 (55°C). (**H**) Chimeras containing the archaeal loop (C2, C3) bind the 3′-flap differently to hFEN1 (compare Figure 4A), but in a manner dependent upon the wedge residue. For (D), results show combined data from rapid quench-flow (*N* = 2, *n* = 4; except for chimera C1, –mut, where *N* = 2, *n* = 3) and manual sampling (*N* = *n* = 4) experiments; for (E), *N* = 4 (hFEN1, chimera C3) or *N* = 6 otherwise. Data used to prepare (D, E) is listed in Supplementary Table S4. ECCD spectra in (F) represent *N* = 42 (DOWN-S-DF1), 5 (+C1, +C2) or 4 (+C3) independent experiments. For (H), *N* = 3. Error bars show SEM.

In addition to the mutagenesis studies above, we further tested the functional importance of loop–arch interactions by constructing three chimeric protein designs (C1–C3, Figure 5A and B), with domain-swapping of either, or both, of the loop and arch regions of *Archaeoglobus fulgidus* FEN1 (AfFEN1) into hFEN1. This strategy allowed us to evaluate whether the loop and arch domains are functionally connected, in which case ‘matched’ domains from the same species should prove optimal. As *A. fulgidus* is a hyperthermophilic organism (optimal growth temperature 83°C), the arch region of AfFEN1 was anticipated to be inherently more structured at 37°C than that of FEN1; and this was reflected in predictions of α-helicity made using the AGADIR (35) algorithm (Figure 5C). Since arch dynamics are evidently important in FEN catalysis (11,31), comparing the human and archaeal sequences should help inform our understanding of this relationship. In fact, evidence from CD spectra did suggest a small increase in overall α-helical content for the chimeras incorporating the archaeal arch domain (C1 and C2; Supplementary Figure S5).

Single-turnover activity of chimeras containing the archaeal arch domain (C1, C2) was at least 200-fold slower than hFEN1, and close to that of AfFEN1 at 37°C, which proved inefficient so far below its normal working temperature (Figure 5D, cyan bars). Chimera C3, containing the human arch domain, essentially matched the activity of hFEN1. Further insight was offered by comparing catalytic efficiency, *k*_cat_/K_M_ (Figure 5E and Supplementary Figure S10A–C). This second-order rate constant reflects both the stability of the enzyme-substrate complex and its rate of decay. Chimera C1, containing the archaeal arch, behaved similarly to AfFEN1. Adding the archaeal loop alongside the arch, as in C2, recovered most of the defect in catalytic efficiency with the change driven primarily by an increase in affinity for substrate. Chimera C3, with only the archaeal loop domain, again proved similar to hFEN1.

Further insights were gained from threading and ECCD results (Figure 5F and Supplementary Figures S9E–G and S10D, E). As anticipated from the above kinetic data, performance in both assays was compromised for C1, but close to that of hFEN1 for C3. In contrast, importantly, chimera C2 was found to thread the 5′-flap fully even though its single turnover reaction rates were slow, and close to those of C1. This result firstly suggested that the ‘matched’ archaeal loop and arch domains in C2 confer a functional advantage in threading compared to incorporating the AfFEN1 arch domain alone (C1); a result in keeping with that noted during kinetic characterization above (higher substrate affinity). But, it also implied that rate defects observed for C2 are due to compromised active site transfer, offering direct evidence that threading and active site positioning are distinct processes.

### An inverse relationship between arch stability and active site transfer revealed by AfFEN1

We presumed the slow rates seen in kinetic experiments with C1, C2 and AfFEN1 at 37°C likely reflected the greater degree of secondary structure possessed by the archaeal arch, indicating an important role for initial disorder in this region for normal FEN1 function. In support of this assumption, threading efficiency of AfFEN1 was found to be strongly temperature-dependent: inefficient with pre-equilibration at 20°C (the usual method), but rescued fully at 55°C (Supplementary Figure S10F). To investigate the relationship of these observations to active site transfer, we proceeded to evaluate AfFEN1 in the ECCD assay at raised temperatures. Using substrate DOWN-S-DF3 (analogous to DOWN-S-DF1 but extended to increase T_m_), a temperature-dependent decrease in substrate-relative peak height was observed consistent with a role for protein flexibility, i.e. conformational dynamics (11), in active site transfer (Figure 5G, cyan bars). This change absolutely required wedge residue Leu47 (equivalent to hFEN1-Leu53), demonstrating that 3′-flap binding also influences active site transfer in AfFEN1 (Figure 5G, orange bars). The process was still suboptimal in AfFEN1 at 55°C, rationalising its inefficiency in the archaeal arch chimeras (as reported above). Nonetheless, the AfFEN1-L47A mutant did prove tenfold slower than the wild-type enzyme at 55°C (Figure 5D), suggesting that this wedge residue plays an equally important role to that of hFEN1-Leu53, with a functional role expected to become increasingly evident as the optimal operating temperature of the enzyme is approached. The apparent requirement seen here for local protein flexibility of the arch, even once ordered, is in agreement with recent structural studies suggesting that short-range motions of the arch might play an important role in positioning the substrate for reaction (10,36).

### The loop–wedge influences substrate binding in hFEN1–AfFEN1 chimeras

We also introduced the equivalent mutation to hFEN1-L53A into the chimeras. As might be expected, these mutations had little effect on the rate of reaction at 37°C because active site transfer is already impeded by the archaeal arch (Figure 5D, orange bars). Nevertheless, differences in substrate binding and reactivity were detectable. Although more subtle than with hFEN1-L53A, small defects were seen in active site transfer in the mutants (Supplementary Figure S8J and K). Also, the archaeal loop of C2 and C3 produced a distinct change in ECCD signal upon binding the 3′-flap (Figure 5H), but this change relied on the presence of the wedge residue (Supplementary Figure S9C). Finally, as was seen for hFEN1-L53A (Figure 4F), C2 and C3 produced more 6-mer product from 5′-single-flap substrates when mutated (Supplementary Figure S9D), indicating that the AfFEN1 wedge residue is also important for binding the substrate in the required double-flap conformation. We thus conclude that the leucine wedge is conserved as the major determinant of FEN1 reaction specificity from mammals to archaea.

### The FEN1 loop–wedge is conserved throughout all domains of life

**Figure 6.**
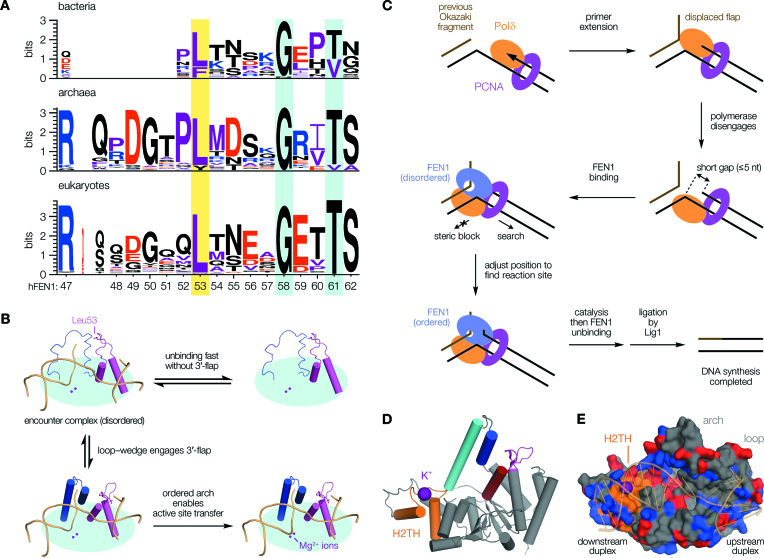
Sequence analysis informs a refined model of cellular FEN1 function. (**A**) The wedge residue (yellow highlight) is present across all domains of life. Other loop positions discussed are also highlighted (cyan). (**B**) Engagement of a single nucleotide 3′-flap by the loop–wedge directly influences active site transfer, occurring over 20 Å away. (**C**) Model for FEN1 flap processing following strand displacement synthesis. Disengagement of DNA polymerase leaves a ‘short gap’ structure (42). Recruitment of FEN1 leads to rapid association with the downstream duplex via its H2TH domain, initiating a short-range ‘search’ to adjust the enzyme's position for reaction. Detection of the upstream (primer) duplex occurs through breaking the terminal base pair and binding the newly-formed 3′-flap, allosterically activating a cascade leading to catalysis. This concept applies equally whether or not any gap re-annealing occurs, requiring a minimum FEN1 translocation of one nucleotide (see Figure 4). It is likely inconsequential at what stage 5′-flap threading occurs during encounter, because this is fast relative to ordering for the short flap-lengths thought to predominate in vivo (56). Additionally, FEN1 and DNA ligase 1 (Lig1)—which seals the nick—cannot be co-resident on PCNA, with their switchover likely controlled through post-translational modifications (57–59). (**D**) hFEN1 structure, as Figure 1D, showing the H2TH domain (orange) and its bound K^+^ ion (purple). (**E**) Surface render of hFEN1 (PDB code: 3Q8K (26)) showing substrate DNA (transparent cartoon) tracking a channel of positively-charged sidechains (blue, with negatively-charged sidechains red). This emphasizes the largely electrostatic nature of the interaction, as has been recognized before (7,60).

Having recognised its importance and conservation in the archaeal and human proteins, we explored whether the ‘wedge strategy’ was common to FEN1 enzymes in general, noting that the loop incorporating this residue meets the formal definition of an omega-loop (Supplementary Figure S6C–E): a protein structural element often associated with recognition processes (37,38). Sequence analysis confirmed the importance of this feature in FENs (Figure 6A and Supplementary Figure S11A) revealing strong conservation of both the wedge residue and C-terminal portion of the loop, which folds to form the core of the 3′-flap pocket upon binding. The wedge and 3′-OH interaction (hFEN1-Thr61) are retained, with an invariant eight amino acid separation punctuated by a highly-conserved glycine (hFEN1-Gly58), suggestive of shared loop geometry. The N-terminal portion of the loop—which forms contacts with the arch (α5)—shows more variation, presumably reflecting contextual adaption of allosteric regulation. It shares common features across eukaryotes and archaea, with small variations in length as noted above. In bacteria, where the FEN peptide exists as the 5′-3′ exonuclease subunit of DNA polymerase I and some differences in regulation might be expected, this part of the loop is truncated—apparently substituted by an extended preceding α-helix—but the 3′-flap binding pocket otherwise strongly resembles that found in other domains of life, and a putative disordered arch region is evident (Supplementary Figure S11B and C). We conclude that ‘loop–wedge motifs’; that is, omega-loops which present a wedge residue in a common orientation, are critical elements in recognition of 3′-flap nucleotides by FENs across all domains of life. It should be clarified that the ‘loop–wedge’ motif we define here—the α2–α3 loop containing the key ‘wedge residue’—is distinct from the ‘hydrophobic wedge’ motif described in earlier structural studies (26), which refers to helices α2 and α3 (including the loop), and is so named because these two α-helices act to ‘wedge open’ the DNA junction, holding it at a sharp angle when bound. The separate loop–wedge mechanism deduced here is reminiscent of other examples, such as *S. pombe* Mag1 (DNA repair) (39) or *S. Solfataricus* Cren7 (chromosome organization) (40), wherein a functionally-important ‘leucine wedge’ residue is inserted between adjacent nucleobases to stabilise the required bound conformation of DNA. However, the positioning of such a key residue within an omega-loop, coupling substrate recognition to allosteric regulation over a significant distance, currently appears to be unique to FEN1.

## DISCUSSION

Our results reveal a central role for allosteric regulation in FEN1, wherein detection and binding of a single-nucleotide 3′-flap are used to control phosphodiester hydrolysis on a different part of the substrate, at an active site over 20 Å away. The 3′-flap interaction causes the α2–α3 loop to order, which then initiates disorder-to-order transition of the arch and subsequent active site transfer of the reacting DNA duplex. The key contact mediating initial substrate verification relies upon a highly-conserved ‘wedge’ residue located on the loop. Once ordered, this forms an omega-loop structure, a feature known to sometimes be associated with allosteric regulation (38). The use of a ‘wedge residue’ interaction to control functional structural transition of the loop has not been described previously in FEN1. Equally importantly, the observation that the ability of the enzyme to induce a 3′-flap depends critically upon the ‘wedge residue’ suggests that this interaction is of primary importance in recognising, and ultimately determining, the required site of reaction. This particular case may be a unique example in that the loop–wedge mediates transmission of structure-specific, but not sequence-specific, information to a remote site through a co-ordinated allosteric cascade, influencing reactivity through control over active site transfer from a distance (Figure 6B).

In the cell, it is essential that FEN1 be recruited to its site of action by the PCNA sliding clamp, since abrogation of their interaction was shown to be lethal in mice (41). It is well established that PCNA co-ordinates the sequential action of replication proteins; but beyond this, it is not precisely clear how correct FEN1 reaction sites are identified. Our present observations indicate that 3′-flap detection is specifically used to couple recognition to catalysis, enabling us to propose a refined model of in vivo FEN1 function in eukaryotes and archaea (Figure 6C).

During lagging strand replication (or LP-BER), the DNA polymerase must disengage after strand displacement synthesis to allow FEN1 access to cleave the flap. Due to the space on the template strand physically occupied by the polymerase, this handover necessarily reveals a ‘short gap’ structure—a poor FEN1 substrate—with a gap length estimated at 2–5 nucleotides (42). It is currently unclear how the double-flap FEN1 substrate then arises.

We propose that, following recruitment by PCNA, FEN1 first binds the downstream duplex via its helix-two-turn-helix domain (H2TH; Figure 6D) and engages in short-range search for an appropriate reaction site through local translocation on DNA. This initial association is non-specific and mostly electrostatic in nature (Figure 6E), facilitating rapid translocation as seen for other H2TH proteins. The reaction site is recognised upon encounter with the upstream duplex, detected by breaking the terminal base pair to place the final nucleotide of the primer strand into the 3′-flap binding pocket (meaning there is no requirement for re-equilibration of the substrate into a 3′-flap conformer). This process induces the single nucleotide 3′-flap of the preferred FEN1 double-flap substrate, allosterically initiating arch ordering, and hence, formation of the more specific and stable functional complex required to activate the catalytic machinery. Biochemical evidence compatible with this model comes from our own kinetics studies (Supplementary Figure S9H), and other reports (1,29,34,36,43), together suggesting that hFEN1—and related family member hEXO1—both bind the downstream duplex DNA of idealised substrates first, followed by repositioning (short-range translocation) of the enzyme prior to catalysis.

Our model is compatible with both exo- and endonucleolytic activities of FEN1, which may both be relevant in vivo (44,45), since the important contacts formed by the ordered arch are with the +1 phosphate (+1P) and template strand (10). Alongside a 3′-flap, these features are common to both substrate types and few additional interactions are made with the 5′-flap (beyond +1P), if present (10).

The proposed mechanism is somewhat reminiscent of the H2TH-containing repair glycosylases (Fpg/Nei family) because a wedge residue (Leu, Phe or Tyr) is employed to recognise the required site of action by all these enzymes (46–48). But, an important functional distinction is seen in that the FEN1–PCNA system must be restricted to short-range search, in contrast to the Fpg/Nei glycosylases that rapidly scan long stretches of DNA for damage.

Alongside an explicit requirement for PCNA interaction to confer sufficient affinity for DNA, the intrinsically disordered regions likely encode this contextual control over FEN1 regulation. With the loop and arch disordered, fast association to give the suggested initial low-specificity (PCNA–FEN1):DNA interaction seems likely, facilitating the ‘search mode’ as explained above. When the target site is encountered, ordering of the 3′-flap binding pocket and subsequent disorder-to-order transition of the arch rapidly and progressively stabilise the complex leading to stalling and tighter binding, now appropriately configured and sufficiently long-lived to proceed with catalysis. These secondary steps will occur only if the favourable additional, specific contacts formed with appropriate substrates occur to offset the entropic penalty associated with ordering; otherwise, binding will be transient.

This kind of substrate verification behaviour has variously been termed ‘kinetic proofreading’ (49) or ‘dock-and-coalesce’ (50); and in a mechanism similar to that proposed here, Dpo4, the translesion polymerase of *S. solfataricus*, is thought to undergo a short-range translocation on DNA in progressing from a non-specific, electrostatically-driven encounter complex to its final, specifically-bound state at the target site (51). In fact, a growing number of systems are now known that exploit multistep binding to achieve selectivity, and for which disorder-to-order transitions provide the underlying mechanism of selection (49,52,53).

In conclusion, our studies reveal an extraordinary level of sophistication in the regulation of FEN1 at the molecular level. The allosteric loop–wedge mechanism we have described provides considerable new insight into how this essential enzyme is regulated, explaining how flap processing is achieved with the high level of accuracy and efficiency necessary to ensure that genome integrity is preserved.

## Supplementary Material

Supplementary Data
