# Supplementary material for: A conserved loop–wedge motif moderates reaction site search and recognition by FEN1
Source: Nucleic Acids Res. 2018 Jun 7;46(15):7858–72. doi: 10.1093/nar/gky506 (PMC6125683; doi:10.1093/nar/gky506)
Supplement: Supplementary Data [file gky506_supplemental_files.pdf]

Supplementary Information Section

A conserved loop–wedge motif moderates reaction site search and recognition by FEN1

Mark J. Thompson,[1] Victoria J. B. Gotham,[1] Barbara Ciani,[1] and Jane A. Grasby[1,*]

[1]Centre for Chemical Biology, Department of Chemistry, Krebs Institute, University of Sheffield, Brook Hill, Sheffield, S3 7HF, UK

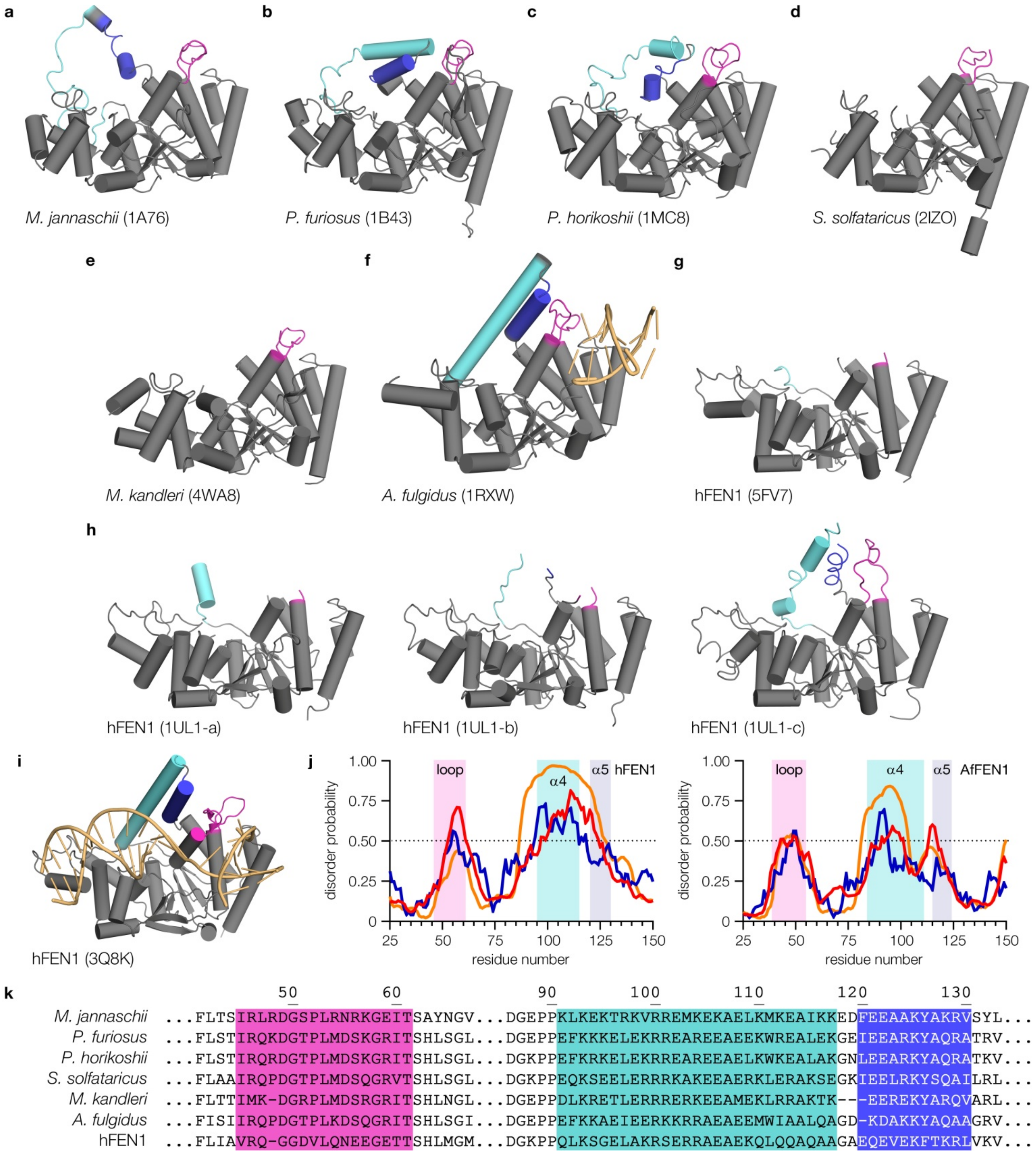

Supplementary Figure S1. Archaeal (1-6) and human (7-9) FEN1 structures show varying degrees of disorder in the loop and arch regions, which order in the presence of bound DNA. Where visible, residues in these regions are coloured as defined in main text Figure 1. (**a–f**) In archaeal FEN1s, the loop and arch appear either unstructured, but with partial formation of secondary structure (**a**,**b**,**c**); or with 'missing' residues, assumed disordered (**d**,**e**). In the presence of duplex DNA containing a 3′-flap, *A. fulgidus* FEN1 (AfFEN1) displays increased order in these regions, with a structured 3′-flap binding pocket and helical arch (**f**) (1). Similar trends are seen in hFEN1 structures (**g–i** and Figure 1d), with varying degrees of disorder evident in the absence of bound DNA (**g**,**h**). Panel (**h**) shows three hFEN1 molecules which were seen bound to the same PCNA homotrimer, but showing different degrees of disorder (8). Interestingly, most of the arch region is assignable—albeit largely unstructured—in the structure with a fully-assigned loop (1UL1-c; right), tentatively supporting the concept that contacts between the loop and α5 may promote structuring of the latter. As with AfFEN1, ordering occurs upon binding of DNA (**i** and Figure 1d). The 3′-flap binding pocket, formed largely from α2–α3 loop residues, is structured and the helical arch has also ordered. (**j**) Three different methods for predicting intrinsic disorder were applied to the hFEN1 and AfFEN1 loop and arch domains, and surrounding regions. In line with expectations, varying degrees of disorder in the loop and arch were predicted by each method employed: IUPred (10,11) (blue), IsUnstruct (12) (orange) and PONDR-FIT (13) (red). (**k**) Sequence alignment of the loop and arch regions of the FEN1s shown in panels (**a–i**). Residues of the α2–α3 loop, α4 and α5 are highlighted according to the same colour scheme used in the panels above, and in main text Figure 1.

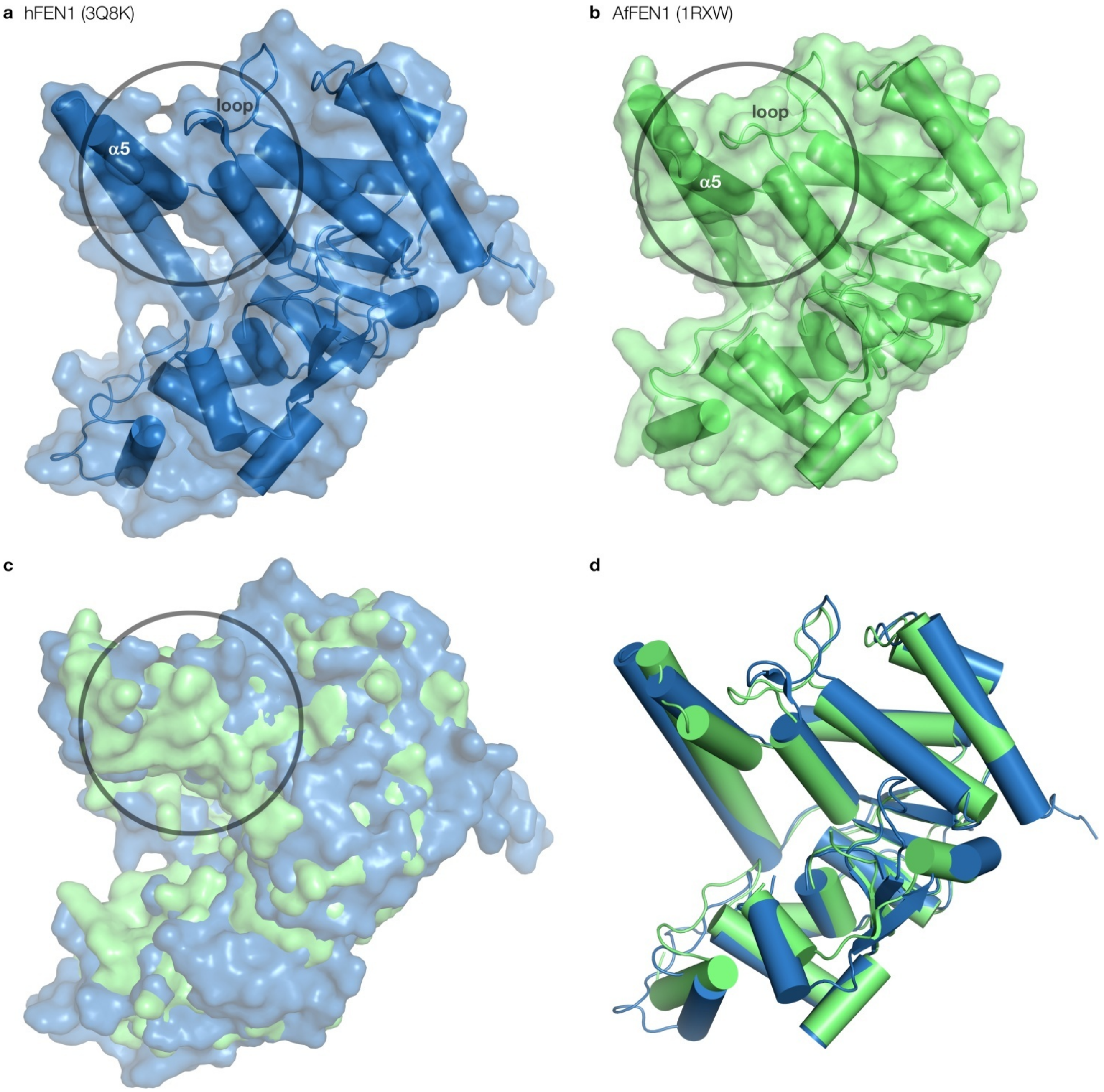

Supplementary Figure S2. Differences are seen in packing between the loop and arch regions when comparing human and archaeal FEN1s. The top panels each show a rendering of the protein surface (transparent) overlaid on a cartoon representation of hFEN1 (left, **a**) or AfFEN1 (right, **b**), aligned to show the proteins in the same orientation. Both proteins are fully ordered, due to being in complex with bound DNA (not shown). The α2–α3 loop ('loop') can clearly be seen to approach α5 more closely in the archaeal protein (right, **b**). In addition, the surface plots indicate a significantly greater contact area between the loop and arch in the archaeal protein in comparison to hFEN1 (compare circled areas in **a**,**b** and note the higher density in this area for the green surface, corresponding to AfFEN1, in the direct overlay, **c**). Alignment of the hFEN1 and AfFEN1 structures, in (**d**), shows otherwise similar protein architecture when in complex with DNA.

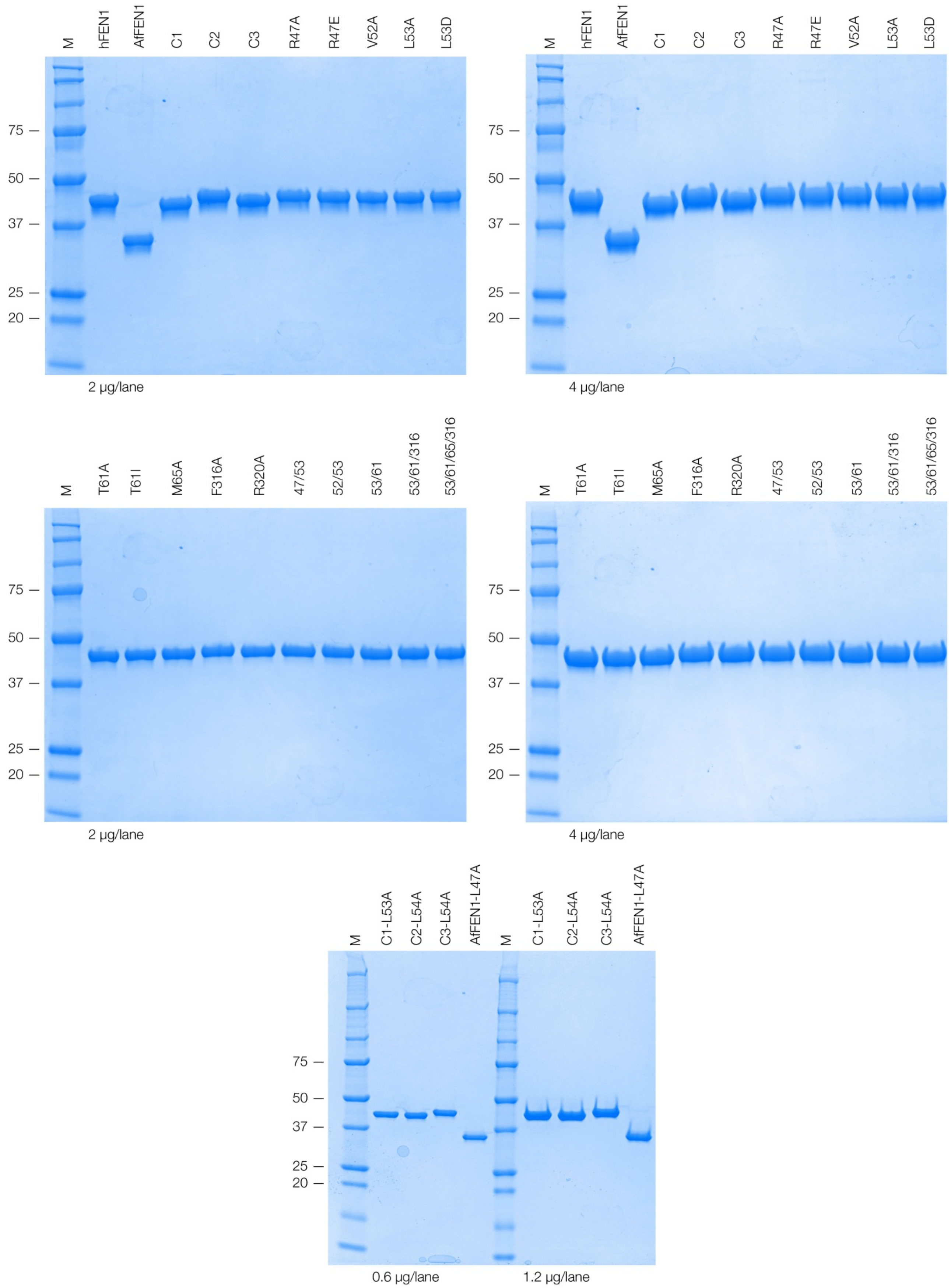

Supplementary Figure S3. SDS-PAGE gel images to illustrate the homogeneity of the purified proteins used in this study. Approximate protein loading per lane is indicated below each gel. 'M' denotes marker lanes, with molecular weights labelled in kDa.

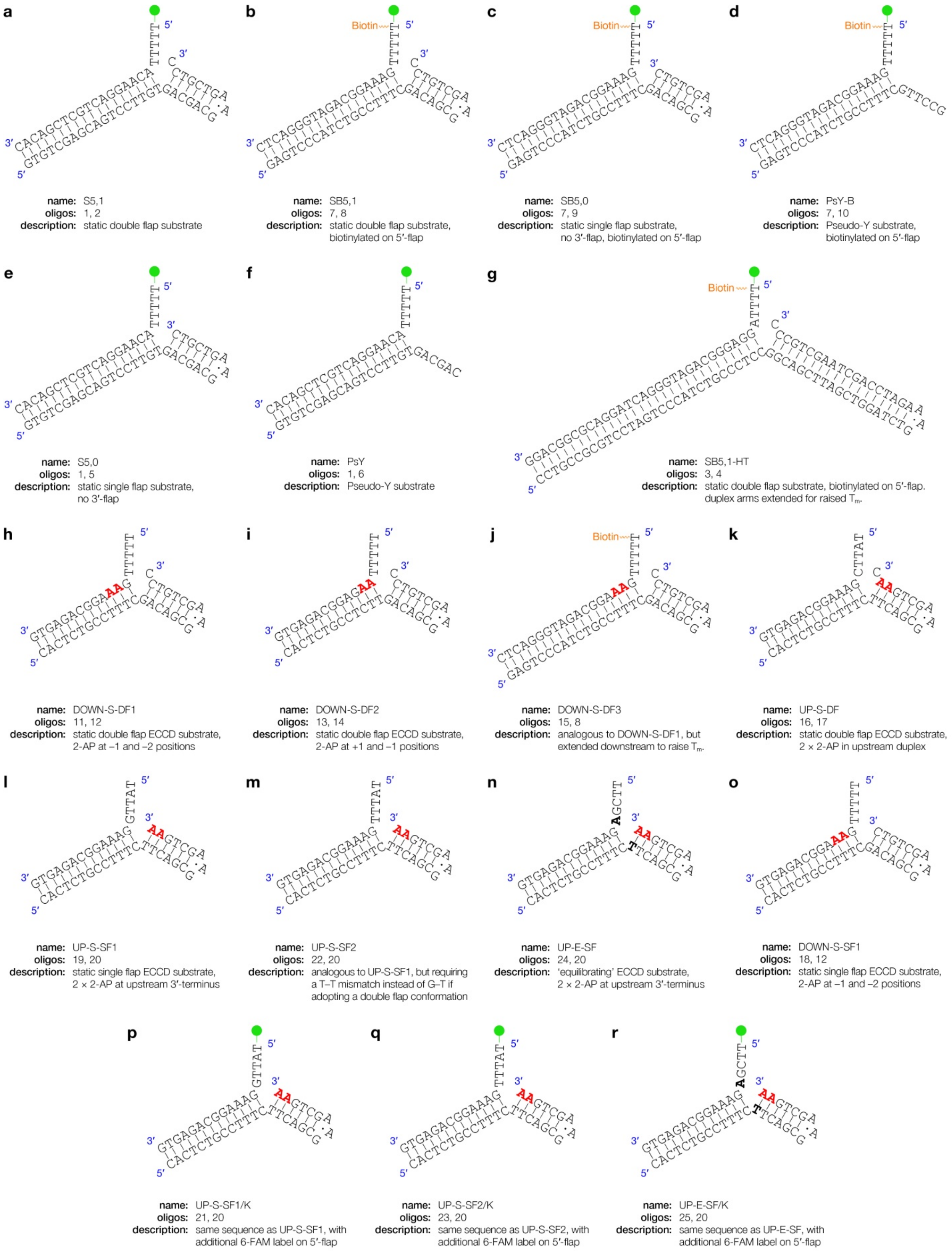

Supplementary Figure S4. Structures of the various DNA substrate constructs used in this study. Green circles represent 6-carboxyfluorescein (6-FAM) labels and red 'A' lettering denotes 2-aminopurine. Bold lettering in panels (**n**) and (**r**) indicates nucleotides which may base pair to form an alternative, double flap conformer. Sequence details for all oligos are provided in Supplementary Table S3.

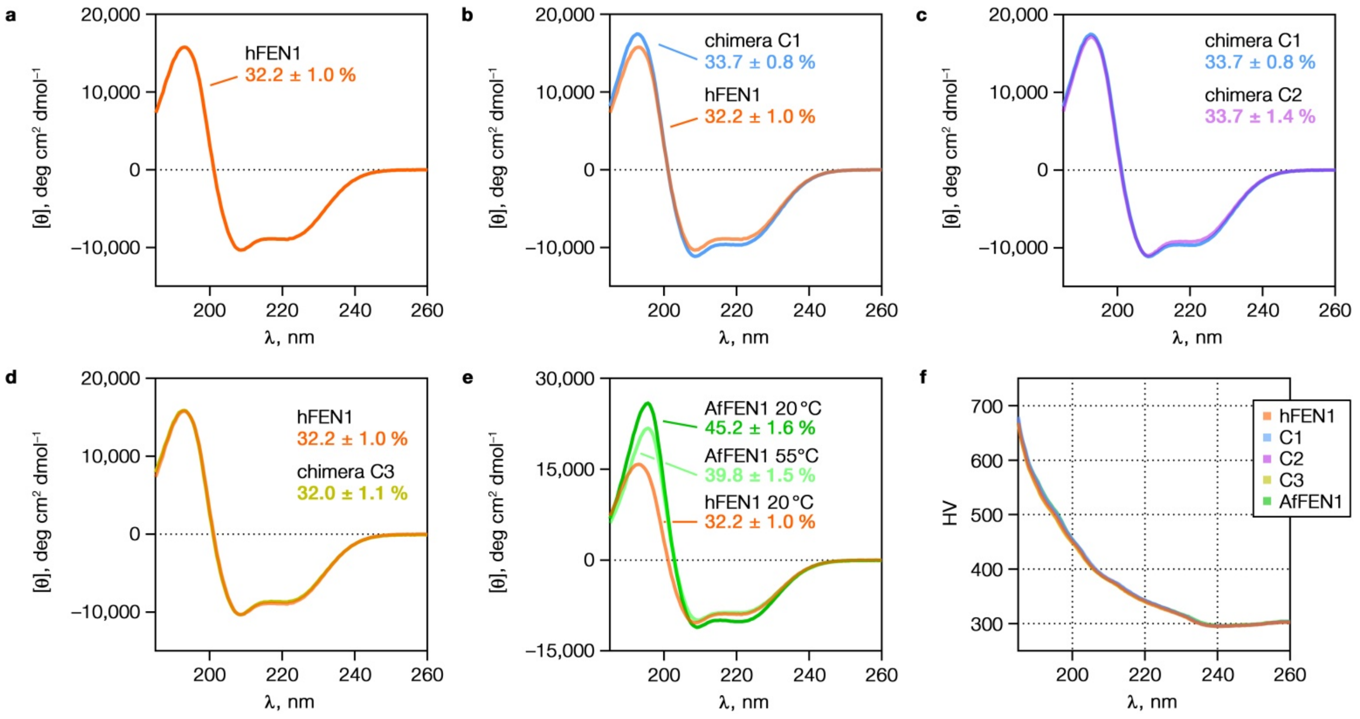

Supplementary Figure S5. CD spectra of hFEN1, AfFEN1 and chimeras C1–C3. All spectra were recorded at 20 °C unless stated, at 3 μM protein. The y-axis for panels (**a**–**e**) is in units of mean residue ellipticity. The percentages shown in (**a**–**e**) correspond to ensemble mean (± standard deviation) for derived α-helical content using the CDSSTR algorithm, as outlined in the methods section. (**a**) Spectrum of hFEN1. (**b**) Chimera C1, containing the more inherently structured AfFEN1 arch region, shows an increase in α-helical content compared to hFEN1, as anticipated. (**c**) Chimera C2, also with the archaeal arch region, shares this trend shown by C1. (**d**) Chimera C3, containing only the AfFEN1 loop region, shows no difference in α-helical content compared to hFEN1, as expected. (**e**) AfFEN1 shows significantly more α-helical content at 20 °C compared to hFEN1, and remains highly structured at 55 °C. (**f**) Overlay of HV plots for all 20 °C spectra, showing that the observed differences are not due to variations in protein concentration. All spectra shown are the global average of three independent experiments, each scanned in triplicate (i.e. N = 3, n = 9).

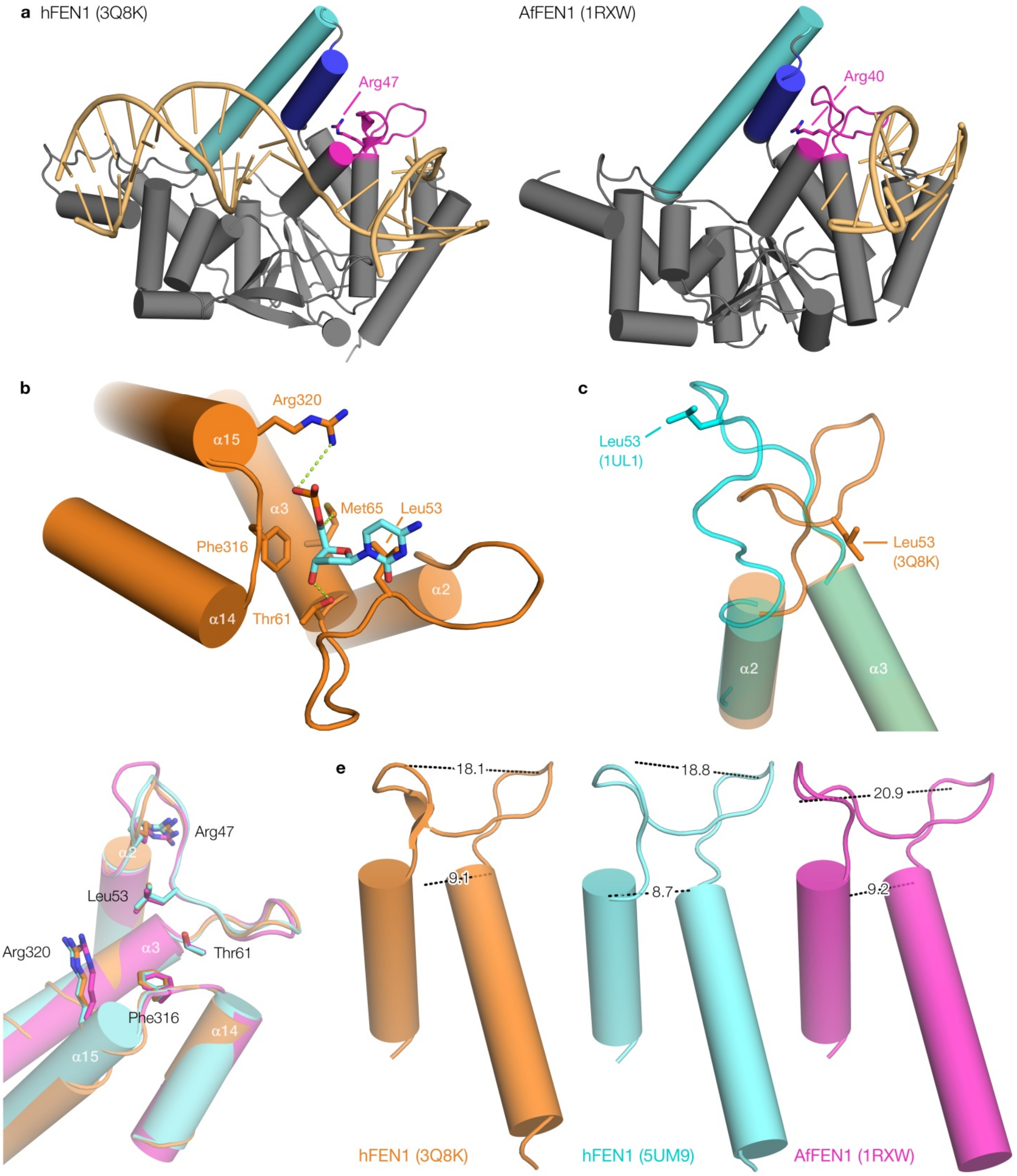

Supplementary Figure S6. Additional views of hFEN1 crystal structures. (**a**) When hFEN1 is bound to DNA (here and Figure 1d), loop residue Arg47 (magenta) is directed towards helix α5 (blue), and is consequently hypothesised to mediate allosteric signalling between these two protein regions. In the related AfFEN1–DNA complex the equivalent residue, Arg40, also adopts a similar orientation. (**b**) Alternative rendering of the structure shown in main text Figure 2a (made using PDB code 5UM9), better emphasising the hydrophobic interactions between Leu53 and the 3′-flap base; and between Phe316 and the 3′-flap nucleoside C3′–5′. (**c**) Overlay of the loop regions from DNA-free hFEN1 (Supplementary Figure 1h, right; PDB code 1UL1, chain Z) and DNA-bound hFEN1 (panel a, left; PDB code 3Q8K) structures indicates that considerable movement of the α2–α3 loop may occur upon binding substrate DNA. The position of key 3′-flap interacting residue Leu53 is shown in each case (sticks). (**d**) Overlay of the 3′-flap binding pocket of hFEN1 (PDB codes 3Q8K (9), orange; and 5UM9 (14), cyan) and AfFEN1 (PDB code 1RXW (1), magenta) in complex with DNA (not shown) reveals very similar overall geometry, and near-identical positioning of conserved amino acid side-chains (sticks). hFEN1 numbering is shown; note that the equivalent residues to Arg47, Leu53, Thr61, Phe316 and Arg320 in AfFEN1 are Arg40, Leu47, Thr55, Phe310 and Arg314, respectively. (**e**) The α2–α3 loop in all three structures meets the classical definition of an omega-loop (Ω-loop) (15,16), because in every case, the C_α–C_α distance between the hinge residues is less than two-thirds of the longest C_α–C_α distance within the loop (distances labelled in Å). Colours are the same as in panel (**d**).

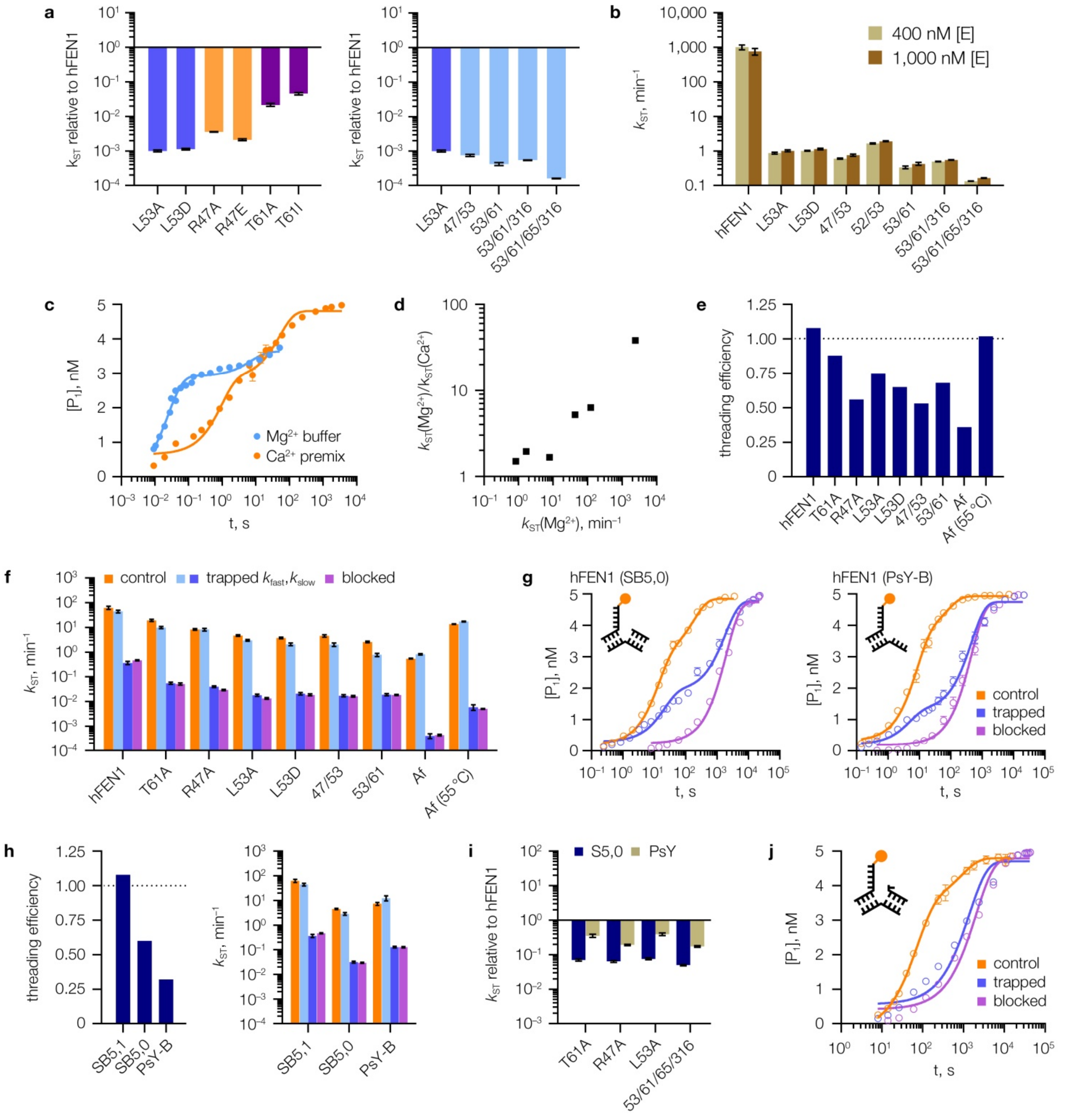

Supplementary Figure S7. Additional data to supplement the results shown in main text Figure 2. (**a**) Mutation of Arg47, Leu53 and Thr61 to residues other than alanine did not produce any significant differences in reactivity (left); and rates with double or triple mutants combining mutation of other residues to alanine alongside Leu53 show no further detriment on introducing these other mutations (right), with the possible exception of the quadruple mutant, which is approximately tenfold slower. (**b**) Single-turnover measurements (k_{ST}) at two enzyme concentrations showed little difference (always within <30%), indicating the maximal rate has likely been reached at the lower [E], and thus suggesting no substantial change in K_M for the mutants. (**c**) If hFEN1 and substrate SB5,1 were premixed in Ca^{2+} buffer—as is the usual method for trapping/blocking experiments—and reaction initiated by adding excess Mg^{2+} (orange curve), the observed rate was considerably slower than that obtained when mixing enzyme and substrate from separate lines, both in Mg^{2+} buffer (the normal single-turnover method; cyan curve). This implied a 'delay effect' in the former case, due to the need for Mg^{2+} to displace Ca^{2+} in the enzyme's active site for reaction to occur. In the case of hFEN1, the time taken for Mg^{2+} to displace Ca^{2+} was evidently long relative to the rate of reaction, leading to an apparent 40-fold rate reduction (the 'delay ratio'). (**d**) Comparing data from analogous experiments for a wider range of enzymes showed that this 'delay ratio' drops off considerably with activity, as might be anticipated, and is not significant relative to the rate of reaction for the slower mutants. (**e**) Threading efficiencies as presented in main text Figure 2f, but for a wider set of enzymes. (**f**) The fast and slow phases of the trapped reaction (cyan, blue) consistently matched the rates with the free substrate (orange) or blocked complex (purple), respectively. (**g**,**h**) Constructs lacking the 3′-flap showed lower threading efficiency, but retained the same trends in rate behaviour as above. (**i**) Relative rate data for reactions with these non-cognate substrates showed similar trends across a set of mutants in which 3′-flap binding is perturbed. (**j**) Quadruple mutant 53/61/65/316 showed only marginal evidence for threading, since the trapped and blocked curves almost overlaid and a 'fast' phase in the trapped case was not clearly evident. Thus, a threading efficiency could not be quantified for this mutant, although the data indicated it must be close to zero. The results presented here combine data from rapid quench-flow and manual sampling techniques, as was appropriate. In (**c**), N = 2, n = 4 (quench-flow technique only). For (**g**,**h**), N = 2, n = 4 (quench-flow phase); and for the manual sampling phase, N = n = 6 (hFEN1 with SB5,1) or N = n = 4 otherwise. In panel (**j**), N = n = 4 (manual sampling). For other panels, replicate information varies between experiments (with minimum N = 2): full details of substrates, measured rate values and replicate information are listed in Supplementary Tables S4 (**a**,**b**) and S5 (**c**–**j**). Error bars in (**c**,**g**,**j**) represent SEM, and in (**a**,**b**,**f**,**h**,**i**) show standard regression errors.

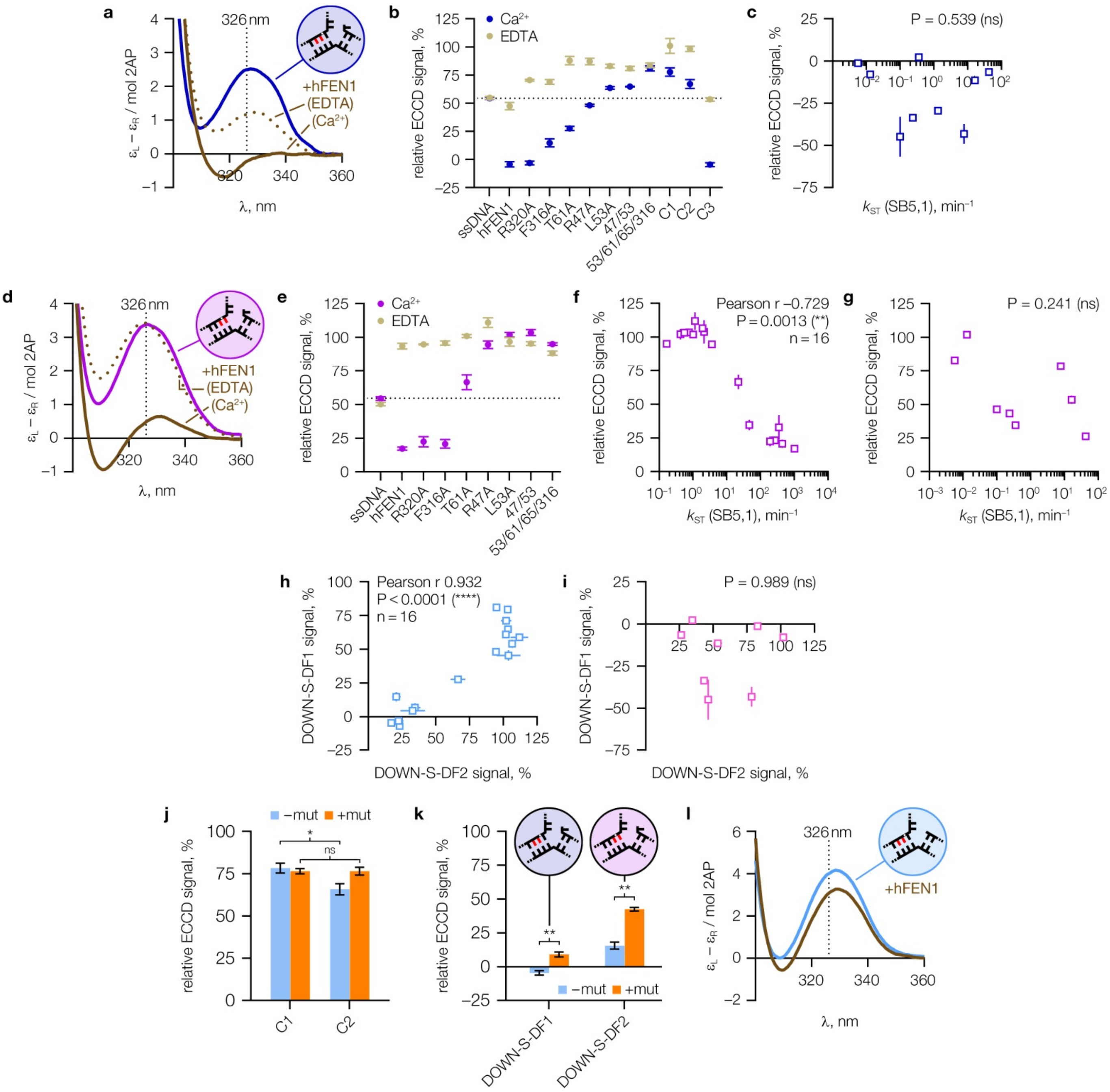

Supplementary Figure S8. Further data from ECCD experiments emphasising the importance of 3′-flap recognition to correct active site transfer of the substrate, and therefore, in promoting the catalytically-competent state. (**a**) Typical set of spectra for substrate DOWN-S-DF1 (2AP bases at –1 and –2 positions as defined in Figs. 1b and 3a), showing results with substrate alone (blue), substrate bound to hFEN1 in Ca^{2+}-containing buffer (brown, solid line) or the same sample after addition of EDTA (i.e. metal ions sequestered from active site; dotted line). (**b**) ECCD peak height at 326 nm (mean ± SEM) for the proteins shown, with substrate DOWN-S-DF1, both in Ca^{2+} buffer (blue) and after addition of EDTA (light brown). For reference, 'ssDNA' shows the signal strength with the labelled flap strand alone. As described in Methods, peak heights are expressed as a percentage relative to substrate peak height from the same experimental run, to control for variations in signal strength between different batches of substrate/oligo. (**c**) Whereas a statistically significant correlation between ECCD signal (DOWN-S-DF1, 326 nm) and enzyme activity was shown for 3′-flap binding mutants (Figure 3c), no such correlation was seen for hFEN1 active site mutants (D34N, Y40A, D86N, K93A, L97P, R100A, R104A, E158Q, D233N). (**d**–**g**) Equivalent data to that in panels (**a**–**c**) for a second substrate, DOWN-S-DF2 (2AP at +1 and –1 positions). Utilising measurements from the same proteins, an analogous correlation to that shown for substrate DOWN-S-DF1 (Figure 3c) is also seen here with 3′-flap binding mutants (**f**): Pearson r –0.729 (95% CI –0.900 to –0.366, R^2 0.532, P 0.0013); but not with active site mutants (**g**). (**h**,**i**) A strong correlation exists between results with the two ECCD substrates for the 3′-flap binding mutants (**h**): Pearson r 0.932 (95% CI 0.810 to 0.976, R^2 0.868, P < 0.0001); but not for the active site mutants (**i**). (**j**) Results with DOWN-S-DF1 suggested a small but meaningful co-operative effect (cyan bars, P 0.022) between the AfFEN1 loop and arch domains (C2), compared to the arch domain alone (C1). This effect was abolished upon mutation of the leucine wedge residue (+mut; orange bars, P 0.999). (**k**) Chimera C3, containing the hFEN1 arch and AfFEN1 loop domains, gives essentially identical ECCD results to hFEN1 ('–mut'; cyan bars) but introduction of the equivalent loop mutation to hFEN1-L53A (C3-L54A; '+mut'; orange bars) led to a significant compromise in active site transfer capability. P values: 0.0052 (DOWN-S-DF1), 0.0028 (DOWN-S-DF2). (**l**) Active site transfer by hFEN1 was prevented for substrate DOWN-S-SF1, analogous to DOWN-S-DF1 with the 3′-flap removed. Panels (**a**,**d**) show representative spectra from N = 13 (DOWN-S-DF1) or N = 10 (DOWN-S-DF2) independent measurements. Data in (**b**,**e**,**h**,**i**) is derived from a minimum of N = 3 independent replicates. In (**c**,**f**,**g**), rates/replicates are as detailed in Supplementary Tables S4 and S6, with a minimum of N = 3 for ECCD results. For (**j**), N = 5 ('–mut') or 3 ('+mut'); for (**k**,**l**), N = 3. All data used in preparing this Figure, with full replicate information (which varies between datasets due to the nature of the experiments involved, and the number of proteins represented), is provided in Supplementary Table S4. Error bars show SEM.

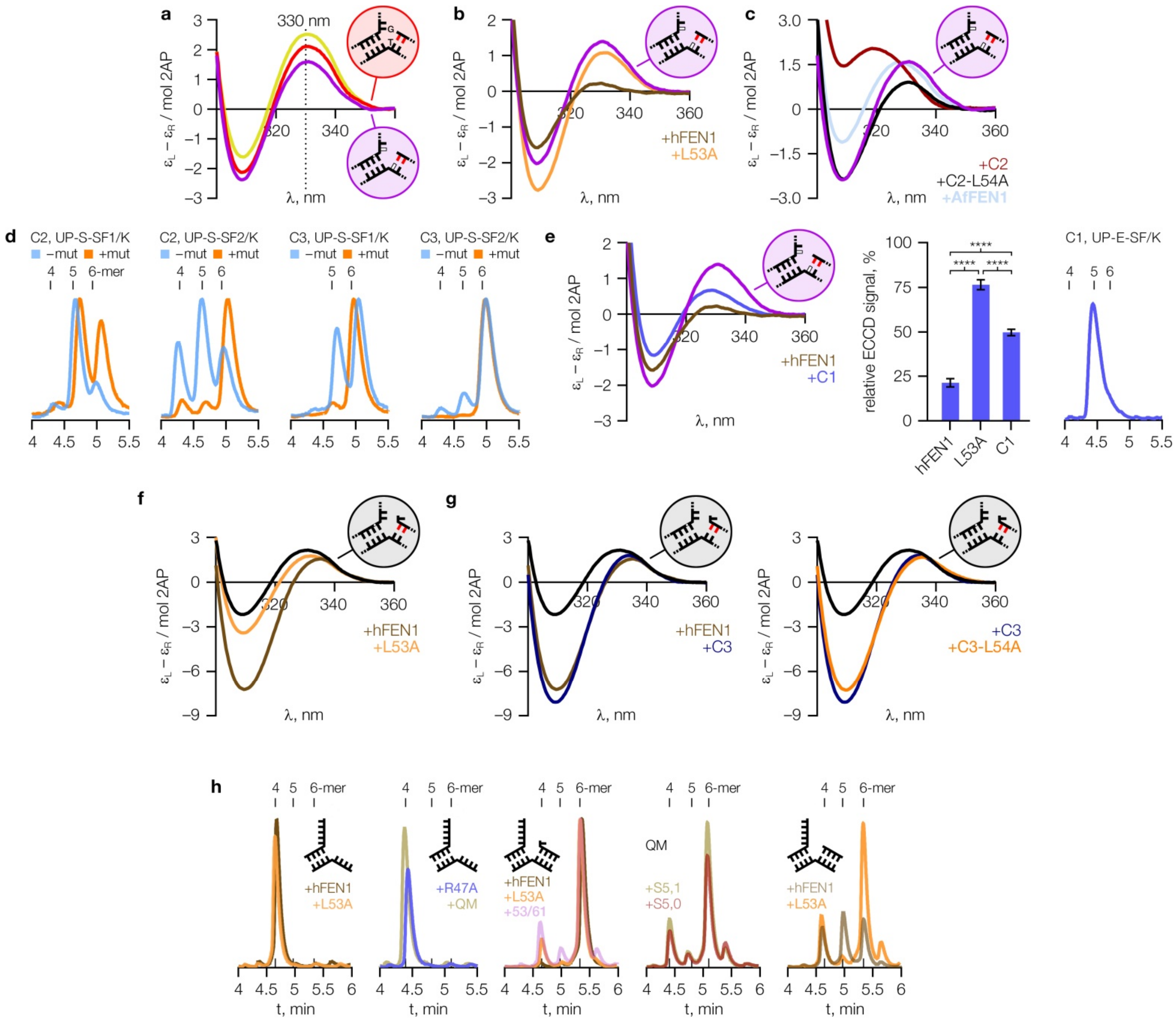

Supplementary Figure S9. Additional results from 'upstream' ECCD, and related, experiments. (**a**) Typical spectra of UP-E-SF (purple), UP-S-SF1 (red) and UP-S-SF2 (sand) indicate the majority of the former is present as the single-flap conformer in solution. (**b**) Main text Figure 4a, adding mutant L53A from the same experimental run. (**c**) AfFEN1 loop-containing chimera C2 displays a similar binding mode, dependent upon the wedge residue, as C3 (main text Figure 5h). (**d**) Mutation of C2-Leu54 or C3-Leu54 did not markedly affect HPLC product profiles with UP-E-SF/K (data not shown), but results with static single-flap substrates showed a noticeable impact, suggesting a subtle but discernible difference in substrate binding. In every case, less 5-mer and more 6-mer product was seen upon mutation, consistent with reaction increasingly in the single-flap form, and therefore with the importance of the leucine wedge in generating the double-flap conformer. With UP-S-SF1/K and UP-S-SF2/K, this necessitates a T–G or T–T mismatch, respectively, at the +1 position. (**e**) Left and centre; chimera C1 (with AfFEN1 arch) is compromised in its ability to generate a 3′-flap conformer from UP-E-SF, but not to the same extent as L53A. P values: 8 × 10^{-8} (hFEN1 vs L53A), 0.000076 (hFEN1 vs C1), 0.000073 (L53A vs C1). Right; since only the 5-mer product was produced from UP-E-SF/K, the double-flap conformer must be formed/bound correctly by C1, suggesting the ECCD results reflect a shorter-lived encounter complex as opposed to binding of the single-flap ('wrong') conformation. If this is a consequence of the low threading efficiency of C1 (Figure 5f), these results further support the proposal that 3′-flap recognition allosterically signals arch ordering, stabilising the initial encounter complex by preventing unthreading. (**f**) We also recorded ECCD spectra with UP-S-DF (pictured), a static double-flap substrate with 2APs in the upstream duplex. The shift seen with hFEN1 was indicative of small conformational changes to the upstream duplex region upon binding. (**g**) In contrast to panel **d** (UP-E-SF), the similar spectra seen for hFEN1, C3 and C3-L54A with UP-S-DF seemingly reported that upstream duplex binding is not appreciably different between these proteins, but rather that the positioning of the 3′-flap nucleotide only varies between hFEN1 and C3. (**h**) Single-turnover product profiles offer evidence for movement of hFEN1 on the substrate after initial encounter, and thereby for translocation/sliding on DNA. Graphs 1 (left) and 2 show a 4-mer is always produced from PsY, not the 6-mer expected based on 5′-flap length, regardless of any 3′-flap binding mutation(s). This suggests initial interaction with the downstream DNA does not immediately place this duplex in the final reactive position, but that adjustment/repositioning is required, reliant on upstream contacts. Graph 3 (centre) shows formation of multiple products, including 4-mer, from S5,1, as 3′-flap contacts are removed (suggesting these contacts are required for correct repositioning of hFEN1 on the substrate). In the extreme case of this, graph 4 shows the 3′-flap binding quadruple mutant (53/61/65/316; 'QM') cannot distinguish between double- and single-flap substrates. It appears that slow reaction occurs with the enzyme positioned variously on the substrate. A similar scenario is seen graph 5 (right; see also Figure 4f), suggesting positioning of the enzyme on S5,0 is not stable in the absence of a 3′-flap. ECCD spectra shown are representative of the following number of replicates: N = 7 (UP-E-SF, **a**), N = 3 (UP-S-SF1, **a**), N = 3 (UP-S-SF2, **a**), N = 5 (hFEN1, **b**), N = 6 (L53A, **b**), N = 3 (all in **c**,**e**), N = 7 (UP-S-DF, **f**), N = 6 (hFEN1, **f**), N = 5 (L53A, **f**), N = 4 (all in **g**). For UP-E-SF summary data (**e**, centre), N = 5 (hFEN1), N = 6 (L53A), or N = 3 (C1). For HPLC chromatograms, N = 2 (**d**,**e**); and for (**h**), from left to right: graph 1, N = 4; graph 2, N = 2; graph 3, N = 5 (except 53/61, N = 4); graph 4, N = 4 (S5,1) or 2 (S5,0); graph 5, N = 4. Full details of the results shown, including replicate information, are listed in Supplementary Tables S6 (ECCD) and S7 (HPLC). Error bars in (**e**) show SEM.

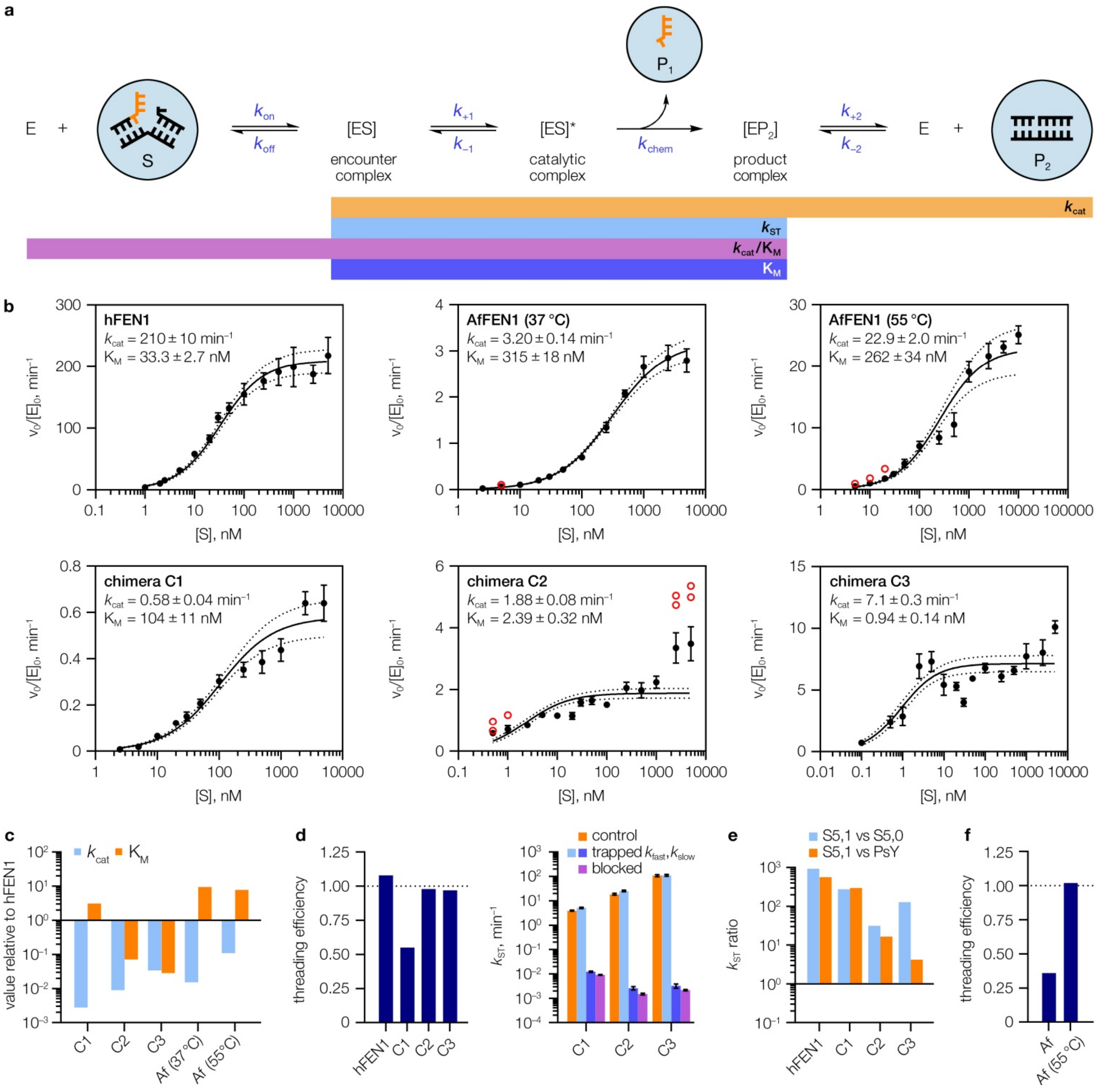

Supplementary Figure S10. Additional data to supplement the results shown in main text Figure 5. (**a**) General FEN1 reaction scheme introducing a definition of terms, and showing which parts of the reaction pathway are assumed to be represented by various catalytic parameters (17). (**b**) Plots of normalised initial rate against substrate concentration for the enzymes detailed in each panel, giving Michaelis–Menten parameters derived in each case. Red circles indicate excluded points which were assigned as outliers by GraphPad Prism. (**c**) Catalytic parameters for the chimeras and AfFEN1 varied widely from those for hFEN1, and did not always show co-variation. (**d**) Threading efficiencies and rate data sets derived from trapping/blocking experiments with the chimeras, analogous to data presented for other protein in main text Figure 2h and Supplementary Figure S7e,f,h. (**e**) Substrate selectivity data analogous to that presented in main text Figure 2e. Incorporation of the archaeal domain(s) in the chimeric proteins produces divergent effects upon selectivity for double-flap (S5,1) over 5′-single-flap (S5,0) and pseudo-Y (PsY) substrates. (**f**) Threading efficiency for AfFEN1 is strongly temperature-dependent, being poor with pre-equilibration at 20 °C (left), but fully rescued when this step is performed at 55 °C (right). In (**b**,**c**), the plots shown are derived from N = 4 replicates (hFEN1, chimera C3) or N = 6 otherwise. In (**d**), for the quench-flow phase: N = 2, n = 4 (C1); N = 5, n = 10 (C2); N = 4, n = 8 (C3); and for the manual sampling phase, N = n = 4 (C1, C3) or 6 (C2). In (**e**), ratios are derived from the rates reported in Supplementary Tables S4 (S5,1) and S5 (S5,0 and PsY), and are listed numerically in Supplementary Table S5. Full details of measured rate data and replicate information can be found in Supplementary Tables S4 (**b**,**c**) and S5 (**d**,**e**); (**f**) is derived from data listed in Supplementary Table S5. Error bars in (**b**) report SEM, and those in (**d**) show standard regression errors.

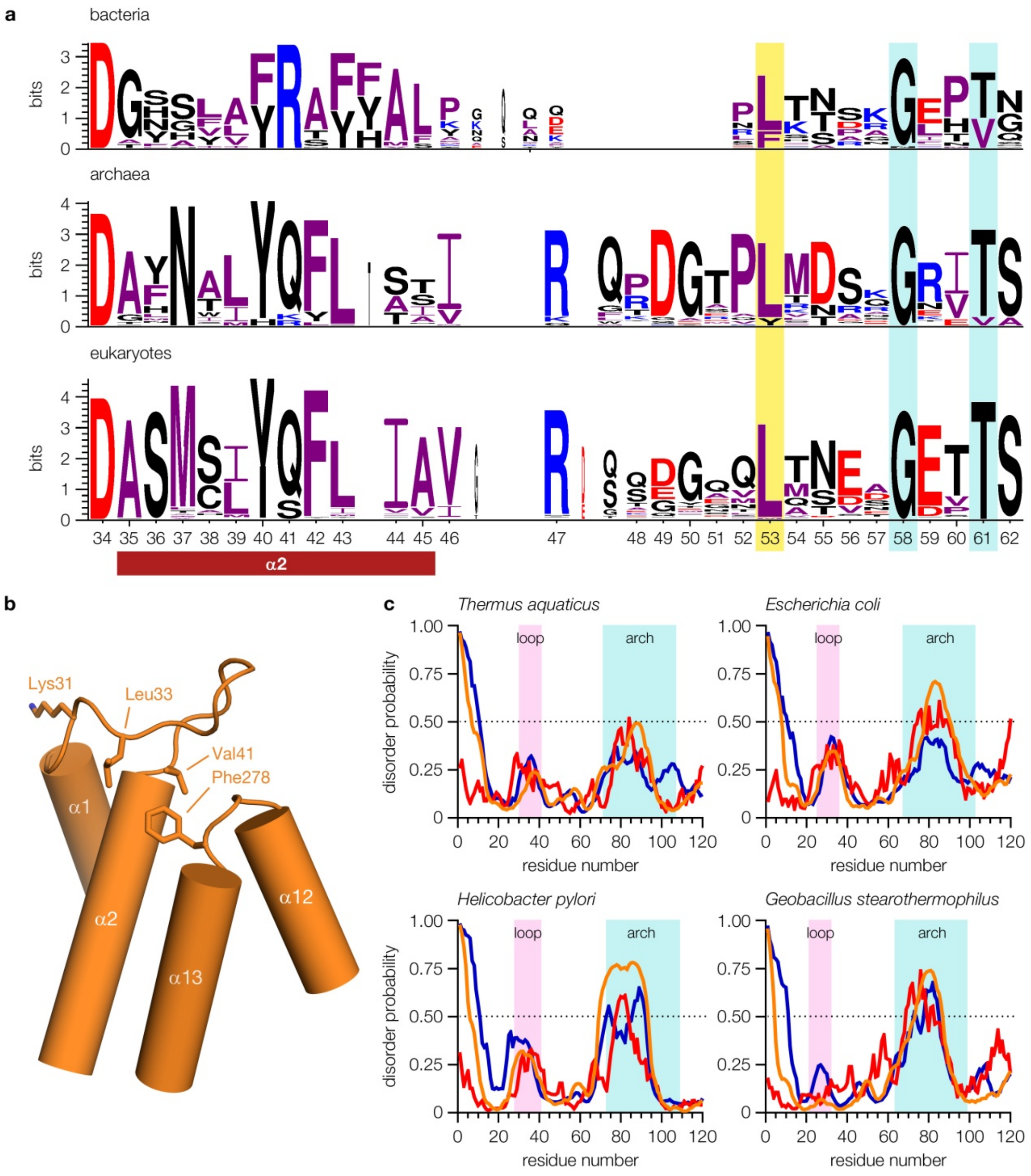

Supplementary Figure S11. Further comparison between FEN proteins across the different domains of life. (**a**) Sequence analysis analogous to that in Figure 6a, expanded to include both the loop region and preceding helix (α2 in hFEN1, comprising the residues indicated). Numbering on x-axis of lower graph indicates hFEN1 residue numbers. Highlighted columns correspond to those discussed in Figure 6, including the wedge residue (yellow). Sequence logos were generated using WebLogo 3 (18,19). (**b**) The 5′-3′ exonuclease domain (FEN1 equivalent peptide) of *Thermus aquaticus* (Taq) polymerase shows a recognisable 3′-flap binding pocket, closely resembling the structure of the hFEN1 and AfFEN1 cases even in the absence of DNA (compare Figure 2a and Supplementary Figure S6b,d). Image prepared using PDB structure 1TAQ (20). (**c**) In addition to the loop–wedge motif shown in panel (**b**), the Taq polymerase structure (1TAQ) contains a disordered region putatively corresponding to the FEN1 arch (residues 71–107). These regions, and the equivalent aligned residues in three other bacterial *polA* sequences, were subjected to intrinsic disorder prediction as described for Supplementary Figure S1j. A disordered region is predicted for each presumed arch domain, with some but variable disorder predicted for the loop (likely reflecting its shorter length in the bacterial case). Plots are coloured according to the algorithm employed, as in Supplementary Figure S1j: IUPred (blue), IsUnstruct (orange) or PONDR-FIT (red).

Supplementary Table S1. Site-directed mutagenesis primers.

Mutagenic Primer Sequences			
**Template**	**Mutation**	**Target**	**Primer Sequences**
hFEN1,<br>L53A	R47A	R47A,<br>R47A/L53A (47/53)	5′-CATCCCCACCCTGGGCAACAGCAATCAGGAAC-3′<br>5′-GTTCCTGATTGCTGTTGCCCAGGGTGGGGATG-3′
hFEN1	R47E	R47E	5′-CACATCCCCACCCTGTTCAACAGCAATCAGGAACTGATAAATGCTC-3′<br>5′-GAGCATTTATCAGTTCCTGATTGCTGTTGAACAGGGTGGGGATGTG-3′
hFEN1	V52A	V52A	5′-CCTCATTCTGCAGCGCATCCCCACCCTGG-3′<br>5′-CCAGGGTGGGGATGCGCTGCAGAATGAGG-3′
hFEN1,<br>T61A	L53A	L53A,<br>L53A/T61A (53/61)	5′-CCCTCCTCATTCTGCGCCACATCCCCACCCTG-3′<br>5′-CAGGGTGGGGATGTGGCGCAGAATGAGGAGGG-3′
hFEN1	L53D	L53D	5′-CACCCTCCTCATTCTGGTCCACATCCCCACCCTGG-3′<br>5′-CCAGGGTGGGGATGTGGACCAGAATGAGGAGGGTG-3′
hFEN1	T61A	T61A	5′-ATCAGGTGGCTGGCGGTCTCACCCTCC-3′<br>5′-GGAGGGTGAGACCGCCAGCCACCTGAT-3′
hFEN1	T61I	T61I	5′-CCATCAGGTGGCTGATGGTCTCACCCTCC-3′<br>5′-GGAGGGTGAGACCATCAGCCACCTGATGG-3′
hFEN1	M65A	M65A	5′-CGGTAGAACATGCCCGCCAGGTGGCTGGTGGT-3′<br>5′-ACCACCAGCCACCTGGCGGGCATGTTCTACCG-3′
hFEN1,<br>T61A	F316A	F316A,<br>T61A/F316A[a]	5′-CGGATTCGCTCCTCAGAGGCCTGCTTTTCACCACACAT-3′<br>5′-ATGTGTGGTGAAAAGCAGGCCTCTGAGGAGCGAATCCG-3′
hFEN1	R320A	R320A	5′-ACCCCACTGCGGATTGCCTCCTCAGAGAACTG-3′<br>5′-CAGTTCTCTGAGGAGGCAATCCGCAGTGGGGT-3′
hFEN1	V52A/L53A	V52A/L53A (52/53)	5′-CCCTCCTCATTCTGCGCCGCATCCCCACCCTGGC-3′<br>5′-GCCAGGGTGGGGATGCGGCGCAGAATGAGGAGGG-3′
T61A/F316A[a]	L53A	L53A/T61A/F316A<br>(53/61/316)	5′-CCCTCCTCATTCTGCGCCACATCCCCACCCTG-3′<br>5′-CAGGGTGGGGATGTGGCGCAGAATGAGGAGGG-3′
L53A/T61A/F316A<br>(53/61/316)	M65A[b]	L53A/T61A/M65A/F316A<br>(53/61/65/316)	5′-GGTAGAACATGCCCGCCAGGTGGCTGGCGG-3′<br>5′-CCGCCAGCCACCTGGCGGGCATGTTCTACC-3′
AfFEN1,<br>chimera C2,<br>chimera C3	Af-L47A	AfFEN1-L47A,<br>chimera C2-L54A,<br>chimera C3-L54A	5′-CCCTGGCTATCTTTCGCCGGTGTACCATCCGG-3′<br>5′-CCGGATGGTACACCGGCGAAAGATAGCCAGGG-3′
chimera C1	C1-L53A	chimera C1-L53A	5′-CACCTTCTTCATTCTGCGCCACATCACCACCCTGAC-3′<br>5′-GTCAGGGTGGTGATGTGGCGCAGAATGAAGAAGGTG-3′

[a]This protein was not expressed, but the construct used as an intermediate in generating the triple (53/61/316) and quadruple (53/61/65/316) mutants. [b]These mutagenic primers were designed with hFEN1-T61A as the template sequence, not wt hFEN1.

Supplementary Table S2. Protein purification conditions.[a]

**Purification Columns**						
	**Columns Run[b]**					
**Protein**	**IMAC**	**AX**	**HEP**	**P/S**	**SEC**	**DESALT**
hFEN1	✓	✓	✓			✓
R47A	✓	✓	✓	✓		✓
R47E	✓	✓	✓			✓
V52A	✓	✓	✓			✓
L53A	✓	✓	✓			✓
L53D	✓	✓	✓			✓
T61A	✓	✓	✓	✓	✓	
T61I	✓	✓	✓	✓	✓	
M65A	✓	✓	✓			✓
F316A	✓	✓	✓			✓
R320A	✓	✓	✓			✓
R47A/L53A (47/53)	✓	✓	✓			✓
V52A/L53A (52/53)	✓	✓	✓			✓
L53A/T61A (53/61)	✓	✓	✓			✓
L53A/T61A/F316A (53/61/316)	✓	✓	✓	✓	✓	
L53A/T61A/M65A/F316A (53/61/65/316)	✓	✓	✓	✓	✓	
AfFEN1[c,d]	✓	✓				✓
AfFEN1-L47A[c]	✓	✓				✓
chimera C1	✓	✓	✓	✓	✓	
chimera C1-L53A	✓	✓	✓			✓
chimera C2	✓	✓	✓	✓	✓	
chimera C2-L54A	✓	✓	✓			✓
chimera C3	✓	✓	✓	✓	✓	
chimera C3-L54A	✓	✓	✓			✓

[a]This Table details which columns were carried out during purification of each individual enzyme. [b]From left to right, the column abbreviations correspond to: IMAC = Ni-NTA column, AX = HiTrap Q FF, HEP = HiPrep Heparin FF 16/10, P/S = HiPrep Phenyl FF (high sub) 16/10, SEC = Sephacryl S-100 HR, DESALT = HiPrep 26/10 desalting. [c]For AfFEN1 and the AfFEN1-L47A mutant, an SDS-PAGE gel of IMAC column fractions was run and care taken that only pure fractions were pooled, since it was anticipated that AfFEN1 might not bind to the heparin column. [d]The heparin column was attempted for AfFEN1, but as suspected, the protein did not bind and was retrieved from the flow-through, then concentrated and taken forward to the desalting step as normal.

Supplementary Table S3. Sequence information for oligonucleotides.

Oligonucleotide Information	
**Oligo No.**	**Sequence**
1	5′-(6-FAM)-TTTTTACAAGGACTGCTCGACAC-3′
2	5′-GTGTCGAGCAGTCCTTGTGACGACGAAGTCGTCC-3′
3	5′-Biotin-5′-(6-FAM)-TTTTAGGAGGGCAGATGGGACTAGGACGCGGCAGG-3′
4	5′-CCTGCCGCGTCCTAGTCCCATCTGCCCTCCGGCAGCTTAGCTGGATCTGAAAGATCCAGCTAAGCTGCCC-3′
5	5′-GTGTCGAGCAGTCCTTGTGACGACGAAGTCGTC-3′
6	5′-GTGTCGAGCAGTCCTTGTGACGAC-3′
7	5′-Biotin-5′-(6-FAM)-TTTTTGAAAGGCAGATGGGACTC-3′
8	5′-GAGTCCCATCTGCCTTTCGACAGCGAAGCTGTCC-3′
9	5′-GAGTCCCATCTGCCTTTCGACAGCGAAGCTGTC-3′
10	5′-GAGTCCCATCTGCCTTTCGTTCCG-3′
11	5′-TTTTTG(2AP)(2AP)AGGCAGAGTG-3′
12	5′-CACTCTGCCTTTCGACAGCGAAGCTGTCC-3′
13	5′-TTTTT(2AP)(2AP)GAGGCAGAGTG-3′
14	5′-CACTCTGCCTCTTGACAGCGAAGCTGTCC-3′
15	5′-Biotin-TTTTTG(2AP)(2AP)AGGCAGATGGGACTC-3′
16	5′-TATTCGAAAGGCAGAGTG-3′
17	5′-CACTCTGCCTTTCTTCAGCGAAGCTG(2AP)(2AP)C-3′
18	5′-CACTCTGCCTTTCGACAGCGAAGCTGTC-3′
19	5′-TATTGGAAAGGCAGAGTG-3′
20	5′-CACTCTGCCTTTCTTCAGCGAAGCTG(2AP)(2AP)-3′
21	5′-(6-FAM)-TATTGGAAAGGCAGAGTG-3′
22	5′-TATTTGAAAGGCAGAGTG-3′
23	5′-(6-FAM)-TATTTGAAAGGCAGAGTG-3′
24	5′-TTCGAGAAAGGCAGAGTG-3′
25	5′-(6-FAM)-TTCGAGAAAGGCAGAGTG-3′

Where present, a terminal 5′-(6-FAM) label was introduced using a separate phosphoramidite (oligos 1, 21, 23 and 25). For oligo 3, the final 5′-nucleotide of the sequence (dT) was incorporated in 6-FAM labelled form, followed by BiotinTEG phosphoramidite (biotin with a tetraethyleneglycol linker). For oligo 7, the 5′-terminus was extended by 6-fluorescein serinol phosphoramidite and BiotinTEG phosphoramidite in sequence to incorporate both 5′-labels. For oligo 15, the 5′-biotin label was incorporated with a bis(ethylene glycol) linker.

Supplementary Table S4. Rate data and replicate information for reactions with ‘static’ double-flap substrates.

**Multiple Turnover Measurements**						
**Enzyme**	**Substrate**	**k_{cat}, min^{-1}**	**K_M, nM**	**k_{cat}/K_M, s^{-1} M^{-1}**	**Replicates (N,n)[a]**	**k_{ST}/k_{cat}**
hFEN1	S5,1	210 ± 10	33.3 ± 2.7	1.05×10^8	BENCH (4,4)	4.86
AfFEN1	S5,1	3.20 ± 0.14	315 ± 18	1.69×10^5	BENCH (6,6)	0.472
AfFEN1 (55 °C)	SB5,1-HT	22.9 ± 2.0	262 ± 34	1.46×10^6	BENCH (6,6)	1.30
chimera C1	S5,1	0.58 ± 0.04	104 ± 11	9.29×10^4	BENCH (6,6)	2.74
chimera C2	S5,1	1.88 ± 0.08	2.39 ± 0.32	1.31×10^7	BENCH (6,6)	2.90
chimera C3	S5,1	7.1 ± 0.3	0.94 ± 0.14	1.26×10^8	BENCH (4,4)	61.3

**Single Turnover Measurements (hFEN1 and mutants), [S] = 5 nM**							
**Enzyme**	**Substrate**	**k_{ST}, min^{-1}**	**[E], nM**	**Replicates (N,n)**	**k_{ST}, min^{-1}**	**[E], nM**	**Replicates (N,n)**
hFEN1	S5,1	*1020 ± 160*	400	QF (2,6)	769 ± 170	1000	QF (2,6)
R47A	S5,1	—	—	—	**3.64 ± 0.08**	1000	QF (2,6)
R47E	S5,1	—	—	—	2.15 ± 0.14	1000	QF (2,4) BENCH (3,3)
V52A	S5,1	270 ± 21	400	QF (2,4)	—	—	—
L53A	S5,1	0.874 ± 0.057	400	QF (2,4) BENCH (4,4)	*1.02 ± 0.06*	1000	QF (2,4) BENCH (4,4)
L53D	S5,1	1.02 ± 0.03	400	BENCH (4,8)	*1.14 ± 0.06*	1000	BENCH (4,8)
T61A	S5,1	—	—	—	22.0 ± 2.1	1000	QF (2,6)
T61I	S5,1	—	—	—	46.6 ± 3.4	1000	QF (2,6)
M65A	S5,1	350 ± 22	400	QF (2,4)	—	—	—
F316A	S5,1	441 ± 36	400	QF (2,4)	—	—	—
R320A	S5,1	192 ± 21	400	QF (2,4)	—	—	—
R47A/L53A (47/53)	S5,1	0.608 ± 0.022	400	BENCH (4,4)	*0.763 ± 0.052*	1000	BENCH (4,4)
V52A/L53A (52/53)	S5,1	1.66 ± 0.05	400	BENCH (4,8)	*1.93 ± 0.06*	1000	BENCH (4,8)
L53A/T61A (53/61)	S5,1	0.339 ± 0.032	400	BENCH (5,5)	*0.428 ± 0.042*	1000	BENCH (5,5)
L53A/T61A/F316A (53/61/316)	S5,1	**0.494 ± 0.009**	400	BENCH (4,4)	***0.555 ± 0.011***	1000	BENCH (4,4)
L53A/T61A/M65A/F316A (53/61/65/316)	S5,1	**0.135 ± 0.002**	400	BENCH (4,4)	***0.167 ± 0.003***	1000	BENCH (4,4)

**Single Turnover Measurements (AfFEN1 and Chimeras)**							
**Enzyme**	**Substrate**	**[S], nM**	**[E], nM**	**k_{ST}, min^{-1} (–mut)[b]**	**Replicates**	**k_{ST}, min^{-1} (+mut)[b]**	**Replicates**
AfFEN1	S5,1	30	3000	**1.51 ± 0.10**	QF (2,4) BENCH (4,4)	4.92 ± 0.92	QF (2,4) BENCH (4,4)
AfFEN1 (55 °C)	SB5,1-HT	30	3000	29.7 ± 1.99	QF (2,4) BENCH (4,4)	3.27 ± 0.34	QF (2,4) BENCH (4,4)
chimera C1	S5,1	10	1000	1.59 ± 0.05	QF (2,3) BENCH (4,4)	2.97 ± 0.08	QF (2,4) BENCH (4,4)
chimera C2	S5,1	0.2	50	**5.45 ± 0.30**	QF (2,4)	2.38 ± 0.23	QF (2,4) BENCH (4,4)
chimera C3	S5,1	0.2	50	435 ± 44	QF (2,4)	218 ± 15	QF (2,4) BENCH (4,4)

Errors stated denote standard errors from nonlinear regression performed in GraphPad Prism. Global fitting to each dataset was used, as outlined in the Methods section. In the case of single turnover measurements a two phase exponential model was used, except for the values highlighted in bold where a single phase model was preferred (see Methods section for additional details). Where more than one enzyme concentration was compared in single turnover measurements, the value used in preparing the Figures (Figure 2c and Supplementary Figure S7a,b) is shown in italics. [a]Data was collected at either short time points (using rapid quench flow; ‘QF’), or longer time points (using manual sampling; ‘BENCH’), or both, as appropriate for each experiment. Replicate information for each technique is given accordingly, where N and n represent the total number of biological and technical replicates, respectively. Multiple turnover measurements were all performed using manual sampling, whereas single turnover experiments required use of either rapid quench flow, manual sampling, or a combination of both techniques. [b]Reactivity of AfFEN1 and chimeras C1–C3 was evaluated with (‘+mut’) or without (‘–mut’) the equivalent mutation to hFEN1-L53A. Specifically, these equivalent mutations are, respectively, AfFEN1-L47A, C1-L53A, C2-L54A and C3-L54A.

Supplementary Table S5. Rate data and replicate information for trapping/blocking and substrate selectivity experiments.

Trapping and Blocking Rates

Enzyme	Substrate	[E], [S]	Control k_{ST}	Trapped k_{fast}	Trapped k_{slow}	Blocked k_{ST}	Replicates (N,n)
hFEN1	SB5,1	500, 5	63.4 ± 9.0	44.9 ± 5.4	0.370 ± 0.052	0.467 ± 0.023	QF (2,4) BENCH (6,6)[a]
hFEN1	SB5,0	1000, 5	4.54 ± 0.26	2.91 ± 0.38	0.0321 ± 0.0028	0.0299 ± 0.0015	QF (2,4) BENCH (4,4)
hFEN1	PsY-B	1000, 5	7.40 ± 1.07	12.6 ± 2.9	0.129 ± 0.010	0.129 ± 0.008	QF (2,4) BENCH (4,4)
hFEN1	SB5,1/EDTA[b]	500, 5	4030 ± 260	3550 ± 560	0.281 ± 0.029	0.265 ± 0.025	QF (2,4) BENCH (6,6)
R47A	SB5,1	500, 5	8.41 ± 0.66	8.29 ± 0.98	0.0403 ± 0.0033	0.0296 ± 0.0017	QF (2,4) BENCH (4,4)
L53A	SB5,1	500, 5	4.76 ± 0.39	3.04 ± 0.22	0.0183 ± 0.0018	0.0136 ± 0.0011	QF (2,4) BENCH (4,4)
L53D	SB5,1	500, 5	3.80 ± 0.28	2.11 ± 0.27	0.0209 ± 0.0023	0.0188 ± 0.0017	QF (2,4) BENCH (6,6)
T61A	SB5,1	500, 5	19.7 ± 2.0	9.98 ± 0.99	0.0555 ± 0.0057	0.0518 ± 0.0046	QF (3,6) BENCH (6,6)
R47A/L53A (47/53)	SB5,1	1000, 5	4.59 ± 0.50	2.05 ± 0.34	0.0173 ± 0.0017	0.0166 ± 0.0012	QF (2,4) BENCH (4,4)
L53A/T61A (53/61)	SB5,1	1000, 5	2.62 ± 0.17	0.790 ± 0.118	0.0192 ± 0.0021	0.0187 ± 0.0008	QF (2,4) BENCH (4,4)
L53A/T61A/M65A/F316A (53/61/65/316)	SB5,1	1000, 5	0.850 ± 0.085	—[c]	**0.0429 ± 0.0037**	0.0291 ± 0.0020	BENCH (4,4)
chimera C1	SB5,1	1000, 10	3.98 ± 0.19	5.18 ± 0.35	0.0125 ± 0.0008	0.00920 ± 0.00026	QF (2,4) BENCH (4,4)
chimera C2	SB5,1	50, 0.2	18.8 ± 1.7	25.5 ± 1.8	0.00261 ± 0.00041	0.00152 ± 0.00013	QF (5,10) BENCH (6,6)
chimera C3	SB5,1	50, 0.2	110 ± 11	112 ± 10	0.00326 ± 0.00064	0.00218 ± 0.00016	QF (4,8) BENCH (4,4)
AfFEN1	SB5,1	3000, 30	0.552 ± 0.023	0.827 ± 0.048	0.000408 ± 0.000092	0.000442 ± 0.000033	QF (2,4) BENCH (4,4)
AfFEN1 (55 °C)	SB5,1-HT	3000, 30	13.9 ± 0.4	17.5 ± 0.5	0.0730 ± 0.0187	0.00508 ± 0.00019	QF (2,4) BENCH (4,4)

Trapping and Blocking Ratios/Controls

Enzyme	Substrate	[E], [S]	Control $\%_{fast}$	Trapped $\%_{fast}$	Threading Efficiency[d]	Mg^{2+} only rate[e]	Replicates (N,n)	Delay Ratio
hFEN1	SB5,1	500, 5	52.6	56.8	1.08	2430 ± 13	QF (2,4)	38.3
hFEN1	SB5,0	1000, 5	59.6	35.6	0.60	—[f]	—[f]	—[f]
hFEN1	PsY-B	1000, 5	72.6	23.3	0.32	—[f]	—[f]	—[f]
hFEN1	SB5,1/EDTA[b]	500, 5	63.7	8.3	0.13	—[f]	—[f]	—[f]
R47A	SB5,1	500, 5	65.2	36.6	0.56	43.9 ± 2.4	QF (2,4)	5.2
L53A	SB5,1	500, 5	65.5	49.1	0.75	**7.97 ± 0.37**	QF (2,4)	1.7
L53D	SB5,1	500, 5	63.6	41.4	0.65	—[f]	—[f]	—[f]
T61A	SB5,1	500, 5	53.4	47.1	0.88	124 ± 8.9	QF (2,4)	6.3
R47A/L53A (47/53)	SB5,1	1000, 5	56.6	29.9	0.53	—[f]	—[f]	—[f]
L53A/T61A (53/61)	SB5,1	1000, 5	56.7	38.8	0.68	—[f]	—[f]	—[f]
L53A/T61A/M65A/F316A (53/61/65/316)	SB5,1	1000, 5	72.4	—[c]	—[c]	1.66 ± 0.13	BENCH (4,4)	2.0
chimera C1	SB5,1	1000, 10	71.8	39.7	0.55	—[f]	—[f]	—[f]
chimera C2	SB5,1	50, 0.2	56.9	55.7	0.98	—[f]	—[f]	—[f]
chimera C3	SB5,1	50, 0.2	61.0	59.1	0.97	—[f]	—[f]	—[f]
AfFEN1	SB5,1	3000, 30	80.3	29.3	0.36	**0.827 ± 0.052**	QF (2,4) BENCH (4,4)	1.5
AfFEN1 (55 °C)	SB5,1-HT	3000,30	87.7	89.2	1.02	29.7 ± 2.0	QF (2,4) BENCH (4,4)	1.7

Rates and Selectivities with S5,0 and PsY Substrates

Enzyme	[E], [S]	k_{ST} (S5,0)	Replicates	k_{ST} (PsY)	Replicates	S5,1 *vs* S5,0	S5,1 *vs* PsY
hFEN1	1000, 5	1.10 ± 0.05	BENCH (8,8)	1.80 ± 0.18	BENCH (7,7)	927	567
R47A	1000, 5	0.0702 ± 0.0042	BENCH (4,4)	0.345 ± 0.015	BENCH (4,4)	51.9	10.6
L53A	1000, 5	0.0834 ± 0.0052	QF (2,3) BENCH (4,4)	0.706 ± 0.064	BENCH (6,6)	12.2	1.4
T61A	1000, 5	0.0774 ± 0.0053	BENCH (4,4)	0.637 ± 0.069	BENCH (4,4)	284	34.5
L53A/T61A/M65A/F316A (53/61/65/316)	1000, 5	0.0546 ± 0.0027	BENCH (4,4)	0.313 ± 0.018	BENCH (4,4)	3.06	0.5
chimera C1	1000, 10	0.00575 ± 0.00026	BENCH (4,4)	0.00530 ± 0.00041	BENCH (4,4)	277	300
chimera C2	50, 0.2	0.173 ± 0.012	BENCH (4,4)	0.328 ± 0.069	BENCH (4,4)	31.5	16.6
chimera C3	50, 0.2	3.39 ± 0.18	QF (2,4) BENCH (4,4)	104 ± 10	QF (2,4) BENCH (4,4)	128	4.2
AfFEN1	3000, 30	0.00329 ± 0.00029	BENCH (4,4)	—[g]	—[g]	459	—[g]

All rates are given in min^{-1} and all concentrations in nM. Errors stated denote standard errors from nonlinear regression performed in GraphPad Prism. Rates are derived from global fitting of each dataset using a one phase exponential model for blocked reactions, and a two phase model for all other cases except where values are highlighted in bold, showing a single phase model was preferred. [a]Data was collected at either short time points (using rapid quench flow; 'QF'), or longer time points (using manual sampling; 'BENCH'), or both; and replicate information for each type of experiment is given accordingly, where N and n represent the total number of biological and technical replicates, respectively. With the exception of hFEN1, blocked reactions were monitored by manual sampling only. [b]Where indicated, samples were premixed in buffer containing 1 mM EDTA instead of 1 mM Ca^{2+}. [c]For the quadruple mutant 53/61/65/316, no significant amount of trapped complex was observed, so a single phase fit was applied to this data set. [d]Threading efficiency, and the methods used to determine these values, are defined in the Methods section. [e]'Mg^{2+} only rate' refers to the rate obtained with the equivalent enzyme and substrate combination under standard single turnover conditions; that is, enzyme and substrate solutions both prepared in Mg^{2+} buffer with reaction initiated by mixing. The 'delay ratio' compares this rate with 'Trapped k_{fast}', to provide a measure of the apparent slowing of the latter due to the need for the excess Mg^{2+} to displace Ca^{2+} from the enzyme's active site when initiating the reaction. [f]These values were not determined. [g]AfFEN1 was essentially unreactive towards the PsY substrate.

Supplementary Table S6. ECCD results summary for 2-aminopurine labelled substrates.[a,f,g]

**‘Downstream’ ECCD Results (Normal Substrates)**									
**Enzyme**	**DOWN-S-DF1 Ca^{2+}, 20 °C**	**DOWN-S-DF1 EDTA, 20 °C**	**N[b]**	**DOWN-S-DF1 Ca^{2+}, 37 °C**	**DOWN-S-DF1 EDTA, 37 °C**	**N**	**DOWN-S-DF2 Ca^{2+}, 20 °C**	**DOWN-S-DF2 EDTA, 20 °C**	**N**
hFEN1	–4.7 ± 2.1	47.1 ± 3.0	13	3.1 ± 3.9	61.2 ± 7.5	3	17.0 ± 1.1	93.6 ± 1.9	10
R47A	48.1 ± 0.9	87.2 ± 2.9	3	–[c]	–	–	94.6 ± 2.8	111 ± 3.5	3
R47E	45.4 ± 3.8	86.9 ± 1.6	3	–	–	–	104 ± 8.8	99.4 ± 2.8	3
R47A/L53A (47/53)	64.8 ± 0.3	81.0 ± 1.8	3	–	–	–	104 ± 2.3	95.4 ± 1.3	3
V52A	–7.2 ± 2.7	52.5 ± 2.6	3	–	–	–	23.2 ± 0.6	102 ± 2.0	3
V52A/L53A (52/53)	54.1 ± 3.1	82.6 ± 1.3	3	–	–	–	107 ± 2.7	96.2 ± 1.0	3
L53A	61.0 ± 2.7	83.1 ± 1.1	4	–	–	–	102 ± 1.7	96.7 ± 3.2	3
L53D	58.8 ± 1.9	79.1 ± 0.5	3	–	–	–	112 ± 6.6	101 ± 5.9	3
L53A/T61A (53/61)	71.2 ± 1.1	80.0 ± 0.8	3	–	–	–	102 ± 3.8	93.8 ± 1.2	3
T61A	27.6 ± 1.9	87.9 ± 3.8	3	–	–	–	66.5 ± 5.5	101 ± 1.1	4
T61I	6.6 ± 3.4	80.0 ± 2.8	3	–	–	–	34.6 ± 4.0	102 ± 4.5	4
M65A	4.4 ± 0.2	54.6 ± 1.8	3	–	–	–	32.9 ± 9.1	103 ± 9.7	4
F316A	14.7 ± 3.5	68.9 ± 2.1	4	–	–	–	20.8 ± 3.1	95.8 ± 1.6	3
R320A	–3.2 ± 1.6	70.5 ± 0.9	3	–	–	–	22.4 ± 3.8	94.8 ± 0.4	3
L53A/T61A/F316A (53/61/316)	79.5 ± 0.5	84.2 ± 0.6	3	–	–	–	103 ± 2.7	95.3 ± 2.6	3
L53A/T61A/M65A/F316A (53/61/65/316)	81.0 ± 2.2	83.8 ± 2.1	3	–	–	–	95.0 ± 1.0	88.3 ± 1.5	3
chimera C1	78.3 ± 2.9	99.5 ± 5.3	5	78.6 ± 3.2	100 ± 2.8	3	–	–	–
chimera C1-L53A	76.5 ± 1.6	83.1 ± 1.0	3	–	–	–	–	–	–
chimera C2	65.8 ± 3.3	97.4 ± 2.0	5	49.3 ± 6.6	95.9 ± 2.4	4	–	–	–
chimera C2-L54A	76.5 ± 2.4	89.4 ± 1.8	3	57.8 ± 2.9	88.8 ± 1.3	3	–	–	–
chimera C3	–6.2 ± 2.0	53.0 ± 1.2	4	2.5 ± 2.4	71.8 ± 2.0	3	15.6 ± 2.6	115 ± 1.2	3
chimera C3-L54A	9.1 ± 1.8	74.3 ± 0.7	3	–	–	–	42.5 ± 1.3	97.3 ± 0.3	3
D34N	–7.9 ± 1.2	60.2 ± 1.6	3	–	–	–	102 ± 1.9	110 ± 0.8	3
Y40A (21)	–43.2 ± 5.8	82.4 ± 3.0	3	–	–	–	78.5 ± 0.9	116 ± 2.7	3
D86N (14)	–29.4 ± 2.2	21.1 ± 3.2	3	–	–	–	–	–	–
K93A (22)	2.3 ± 2.2	94.6 ± 3.8	3	–	–	–	34.5 ± 1.4	103 ± 1.3	3
L97P (22)	–1.3 ± 0.9	95.8 ± 2.5	3	–	–	–	83.0 ± 2.2	124 ± 0.6	3
R100A (22)	–44.8 ± 11.9	86.2 ± 9.9	4	–	–	–	46.3 ± 1.3	110 ± 4.3	3
R104A (14)	–11.4 ± 2.0	80.6 ± 4.2	4	–	–	–	53.4 ± 3.2	113 ± 7.6	4
E158Q	–33.7 ± 0.6	74.0 ± 1.3	3	–	–	–	43.2 ± 1.8	106 ± 3.3	3
D233N (14)	–6.5 ± 0.2	17.9 ± 0.1	3	–	–	–	26.3 ± 2.3	74.5 ± 1.5	3
**‘Downstream’ ECCD Results (Extended Substrate DOWN-S-DF3)[d]**									
**Enzyme**	**DOWN-S-DF3 Ca^{2+}, 20 °C**	**DOWN-S-DF3 EDTA, 20 °C**	**N**	**DOWN-S-DF3 Ca^{2+}, 37 °C**	**DOWN-S-DF3 EDTA, 37 °C**	**N**	**DOWN-S-DF3 Ca^{2+}, 55 °C**	**DOWN-S-DF3 EDTA, 55 °C**	**N**
hFEN1[e]	2.7	42.5	1	–1.3	53.2	1	–	–	–
AfFEN1	89.8 ± 0.8	101 ± 1.6	3	62.1 ± 2.4	96.0 ± 0.6	3	47.1 ± 2.8	94.1 ± 3.6	3
AfFEN1-L47A	96.8 ± 1.3	98.4 ± 2.4	3	98.6 ± 2.8	102 ± 0.7	3	97.3 ± 3.1	97.6 ± 4.5	3
**‘Upstream’ ECCD Results**									
**Enzyme**	**UP-E-SF Ca^{2+}, 20 °C**	**UP-E-SF EDTA, 20 °C**	**N**	**UP-S-SF1 Ca^{2+}, 20 °C**	**UP-S-SF1 EDTA, 20 °C**	**N**	**UP-S-SF2 Ca^{2+}, 20 °C**	**UP-S-SF2 EDTA, 20 °C**	**N**
hFEN1	21.4 ± 2.3	70.2 ± 8.1	5	42.9 ± 2.8	73.3 ± 3.6	4	34.9 ± 0.4	68.6 ± 4.7	4
R47A	57.2 ± 4.4	84.5 ± 6.4	3	–	–	–	–	–	–
L53A	76.5 ± 2.7	92.7 ± 6.6	6	79.1 ± 3.0	89.2 ± 1.0	4	82.3 ± 1.7	91.8 ± 1.5	4
R47A/L53A (47/53)	83.1 ± 0.8	92.8 ± 5.4	3	–	–	–	–	–	–
chimera C1	49.8 ± 1.8	77.6 ± 3.6	3	–	–	–	–	–	–

[a]All results are derived from peak height at 326 nm (‘DOWN-’ substrates) or 330 nm (‘UP-’ substrates), expressed as percentages (mean ± SEM) relative to substrate signal on the same experimental run. This is to control for variations in signal strength between different substrate/oligo batches, as explained in Methods. [b]‘N’ represents the number of independent measurements for each enzyme/substrate combination. [c]A dash indicates values not determined. [d]Substrate DOWN-S-DF3 is analogous to DOWN-S-DF1 (2AP bases at –1 and –2 positions) but with an extended ‘downstream’ duplex to increase T_m; see Supplementary Figure S4 for details of all substrate structures and oligo sequences. [e]Control measurements were made once each at 20 °C and 37 °C, with hFEN1 and DOWN-S-DF3, to validate that the substrate behaved as expected (i.e. analogously to DOWN-S-DF1). [f]The results for hFEN1 with substrate DOWN-S-SF1 were: 74.6 ± 4.1% (Ca^{2+}), 88.6 ± 4.2% (EDTA), N = 3; stated here but not tabulated otherwise. [g]In preparing Supplementary Figure S6 (panels c,g,i), reported rate data was used for Y40A, D86N, K93A, L97P, R100A, R104A and D233N. Single turnover experiments at 400 nM [E], 5 nM [S]—as described in the Methods section—gave rate values of 0.013 ± 0.0004 min^{-1} (N = 4) for D34N, and 0.24 ± 0.01 min^{-1} (N = 5) for E158Q.

Supplementary Table S7. Conditions associated with HPLC chromatograms.

**Quenched Reaction Details**						
**Figure[a]**	**Enzyme**	**Substrate**	**[E], [S], nM**	**Time Point, hh:mm:ss**	**Conversion, %[b]**	**N[c]**
4c	hFEN1	UP-E-SF/K	400, 5	00:00:08	66.8	4
4c	L53A	UP-E-SF/K	1000, 5	00:03:20	75.0	2
4c	R47A	UP-E-SF/K	1000, 5	00:02:40	69.8	2
4c	R47A/L53A (47/53)	UP-E-SF/K	1000, 5	00:06:40	72.5	2
4f	hFEN1	UP-S-SF1/K	1000, 5	00:01:00	77.9	4
4f	L53A	UP-S-SF1/K	1000, 5	00:20:00	92.5	4
4f	hFEN1	UP-S-SF2/K	1000, 5	00:01:00	79.8	4
4f	L53A	UP-S-SF2/K	1000, 5	00:20:00	97.4	4
S9d	chimera C2	UP-S-SF1/K	100, 0.5	00:20:00	84.4	2
S9d	chimera C2-L54A	UP-S-SF1/K	100, 0.5	00:20:00	62.3	2
S9d	chimera C2	UP-S-SF2/K	100, 0.5	00:20:00	80.5	2
S9d	chimera C2-L54A	UP-S-SF2/K	100, 0.5	00:20:00	68.0	2
S9d	chimera C3	UP-S-SF1/K	100, 0.5	00:00:50	69.7	2
S9d	chimera C3-L54A	UP-S-SF1/K	100, 0.5	00:01:40	65.4	2
S9d	chimera C3	UP-S-SF2/K	100, 0.5	00:01:40	81.3	2
S9d	chimera C3-L54A	UP-S-SF2/K	100, 0.5	00:01:40	74.6	2
S9d	chimera C1	UP-E-SF/K	1000, 10	00:01:40	67.2	2
S9h	hFEN1	S5,1	400, 5	00:00:06	70.8	5
S9h	L53A	S5,1	1000, 5	00:06:49	82.5	5
S9h	L53A/T61A (53/61)	S5,1	1000, 5	00:16:12	87.2	4
S9h	L53A/T61A/M65A/F316A (53/61/65/316; QM)	S5,1	1000, 5	01:22:40	96.8	4
S9h	hFEN1	PsY	1000, 5	00:03:51	50.3	4
S9h	L53A	PsY	1000, 5	00:09:49	52.1	4
S9h	R47A	PsY	1000, 5	00:10:05	51.0	2
S9h	L53A/T61A/M65A/F316A (53/61/65/316; QM)	PsY	1000, 5	00:22:10	56.9	2
S9h	hFEN1	S5,0	1000, 5	00:06:17	73.6	4
S9h	L53A	S5,0	1000, 5	01:23:10	92.6	4
S9h	L53A/T61A/M65A/F316A (53/61/65/316; QM)	S5,0	1000, 5	01:03:30	88.1	2

[a]In Figure references, the 'S' prefix denotes reference to a Supplementary Figure. [b]Total conversion at the time point sampled; i.e. where more than one product was formed, the sum of all product peaks is reported. [c]The number of independent experiments of which the chromatogram presented is representative. Note that these examples were performed independently of kinetics (k_{ST}) experiments.

References for Supplementary Information Section

1. Chapados, B.R., Hosfield, D.J., Han, S., Qiu, J., Yelent, B., Shen, B. and Tainer, J.A. (2004) Structural basis for FEN-1 substrate specificity and PCNA-mediated activation in DNA replication and repair. *Cell*, **116**, 39-50.

2. Dore, A.S., Kilkenny, M.L., Jones, S.A., Oliver, A.W., Roe, S.M., Bell, S.D. and Pearl, L.H. (2006) Structure of an archaeal PCNA1-PCNA2-FEN1 complex: elucidating PCNA subunit and client enzyme specificity. *Nucleic Acids Res.*, **34**, 4515-4526.

3. Hosfield, D.J., Mol, C.D., Shen, B. and Tainer, J.A. (1998) Structure of the DNA repair and replication endonuclease and exonuclease FEN-1: coupling DNA and PCNA binding to FEN-1 activity. *Cell*, **95**, 135-146.

4. Hwang, K.Y., Baek, K., Kim, H.-Y. and Cho, Y. (1998) The crystal structure of flap endonuclease-1 from *Methanococcus jannaschii*. *Nat. Struct. Mol. Biol.*, **5**, 707-713.

5. Matsui, E., Musti, K.V., Abe, J., Yamasaki, K., Matsui, I. and Harata, K. (2002) Molecular structure and novel DNA binding sites located in loops of flap endonuclease-1 from *Pyrococcus horikoshii*. *J. Biol. Chem.*, **277**, 37840-37847.

6. Shah, S., Dunten, P., Stiteler, A., Park, C.K. and Horton, N.C. (2015) Structure and specificity of FEN-1 from *Methanopyrus kandleri*. *Proteins: Struct., Funct., Bioinf.*, **83**, 188-194.

7. Exell, J.C., Thompson, M.J., Finger, L.D., Shaw, S.J., Debreczeni, J., Ward, T.A., McWhirter, C., Sioberg, C.L.B., Molina, D.M., Abbott, W.M. *et al.* (2016) Cellularly active *N*-hydroxyurea FEN1 inhibitors block substrate entry to the active site. *Nat. Chem. Biol.*, **12**, 815-821.

8. Sakurai, S., Kitano, K., Yamaguchi, H., Hamada, K., Okada, K., Fukuda, K., Uchida, M., Ohtsuka, E., Morioka, H. and Hakoshima, T. (2005) Structural basis for recruitment of human flap endonuclease 1 to PCNA. *EMBO J.*, **24**, 683-693.

9. Tsutakawa, Susan E., Classen, S., Chapados, Brian R., Arvai, A.S., Finger, L.D., Guenther, G., Tomlinson, Christopher G., Thompson, P., Sarker, Altaf H., Shen, B. *et al.* (2011) Human flap endonuclease structures, DNA double-base flipping, and a unified understanding of the FEN1 superfamily. *Cell*, **145**, 198-211.

10. Dosztányi, Z., Csizmok, V., Tompa, P. and Simon, I. (2005) IUPred: web server for the prediction of intrinsically unstructured regions of proteins based on estimated energy content. *Bioinformatics*, **21**, 3433-3434.

11. Dosztányi, Z., Csizmók, V., Tompa, P. and Simon, I. (2005) The pairwise energy content estimated from amino acid composition discriminates between folded and intrinsically unstructured proteins. *J. Mol. Biol.*, **347**, 827-839.

12. Lobanov, M.Y. and Galzitskaya, O.V. (2011) The Ising model for prediction of disordered residues from protein sequence alone. *Phys. Biol.*, **8**, 035004.

13. Xue, B., Dunbrack, R.L., Williams, R.W., Dunker, A.K. and Uversky, V.N. (2010) PONDR-FIT: a meta-predictor of intrinsically disordered amino acids. *Biochim. Biophys. Acta Protein Proteomics*, **1804**, 996-1010.

14. Tsutakawa, S.E., Thompson, M.J., Arvai, A.S., Neil, A.J., Shaw, S.J., Algasaier, S.I., Kim, J.C., Finger, L.D., Jardine, E., Gotham, V.J.B. *et al.* (2017) Phosphate steering by flap endonuclease 1 promotes 5′-flap specificity and incision to prevent genome instability. *Nat. Commun.*, **8**, 15855.

15. Fetrow, J.S. (1995) Omega loops: nonregular secondary structures significant in protein function and stability. *FASEB J.*, **9**, 708-717.

16. Leszczynski, J. and Rose, G. (1986) Loops in globular proteins: a novel category of secondary structure. *Science*, **234**, 849-855.

17. Finger, L.D., Blanchard, M.S., Theimer, C.A., Sengerová, B., Singh, P., Chavez, V., Liu, F., Grasby, J.A. and Shen, B. (2009) The 3′-flap pocket of human flap endonuclease 1 Is critical for substrate binding and catalysis. *J. Biol. Chem.*, **284**, 22184-22194.

18. Crooks, G.E., Hon, G., Chandonia, J.-M. and Brenner, S.E. (2004) WebLogo: a sequence logo generator. *Genome Res.*, **14**, 1188-1190.

19. Schneider, T.D. and Stephens, R.M. (1990) Sequence logos: a new way to display consensus sequences. *Nucleic Acids Res.*, **18**, 6097-6100.

20. Kim, Y., Eom, S.H., Wang, J., Lee, D.S., Suh, S.W. and Steitz, T.A. (1995) Crystal structure of *Thermus aquaticus* DNA polymerase. *Nature*, **376**, 612-616.

21. Algasaier, S.I., Exell, J.C., Bennet, I.A., Thompson, M.J., Gotham, V.J.B., Shaw, S.J., Craggs, T.D., Finger, L.D. and Grasby, J.A. (2016) DNA and protein requirements for substrate conformational changes necessary for human flap endonuclease-1-catalyzed reaction. *J. Biol. Chem.*, **291**, 8258-8268.

22. Patel, N., Exell, J.C., Jardine, E., Ombler, B., Finger, L.D., Ciani, B. and Grasby, J.A. (2013) Proline scanning mutagenesis reveals a role for the flap endonuclease-1 helical cap in substrate unpairing. *J. Biol. Chem.*, **288**, 34239-34248.
